# Trait to gene analysis reveals that allelic variation in three genes determines seed vigour

**DOI:** 10.1111/nph.14102

**Published:** 2016-07-19

**Authors:** Karl Morris, Guy C. Barker, Peter G. Walley, James R. Lynn, William E. Finch‐Savage

**Affiliations:** ^1^ School of Life SciencesWellesbourne CampusWarwick UniversityWellesbourneWarwickCV35 9EFUK; ^2^ Functional and Comparative GenomicsInstitute of Integrative BiologyUniversity of LiverpoolLiverpoolL69 7ZBUK

**Keywords:** abscisic acid (ABA), allelic variation, *Arabidopsis*, *Brassica oleracea*, germination, QTL analysis, seed vigour

## Abstract

- Predictable seedling establishment is essential for resource‐efficient and cost‐effective crop production; it is widely accepted as a critically important trait determining yield and profitability. Seed vigour is essential to this, but its genetic basis is not understood.
- We used natural variation and fine mapping in the crop *Brassica oleracea* to show that allelic variation at three loci influence the key vigour trait of rapid germination. Functional analysis in both *B. oleracea* and the model *Arabidopsis* identified and demonstrated activity of genes at these loci.
- Two candidate genes were identified at the principal Speed of Germination QTL (*SOG1*) in *B. oleracea*. One gene *BoLCVIG2* is a homologue of the alternative‐splicing regulator (*AtPTB1*). The other gene *BoLCVIG1* was unknown, but different alleles had different splice forms that were coincident with altered abscisic acid (ABA) sensitivity. We identified a further QTL, Reduced ABscisic Acid 1 (*RABA1*) that influenced ABA content and provide evidence that this results from the activity of a homologue of the ABA catabolic gene *AtCYP707A2* at this locus.
- Lines containing beneficial alleles of these three genes had greater seed vigour. We propose a mechanism in which both seed ABA content and sensitivity to it determines speed of germination.

## Introduction

Predictable seedling establishment is essential for crop production to be both resource efficient and cost effective and is therefore widely accepted as a critically important trait for farmers and for food security. Seedling establishment is dependent upon high seed vigour, which is required to cope with the variable seedbed conditions experienced in agriculture (Finch‐Savage, 1995). However, the causes of variation in seed vigour are not fully understood and in particular its genetic basis is unclear (Finch‐Savage & Bassel, 2016). To have an impact on this problem it was essential to study vigour traits that have reproducible effects in agricultural practice. These traits were examined previously in extensive field and laboratory studies to develop an approach to identifying genes that regulate seed vigour in *Brassica oleracea* (Finch‐Savage *et al*., 2010) This work suggested that a strategy of stress avoidance, through rapid germination and subsequent rapid pre‐emergence seedling growth, has an advantage in agriculture over stress tolerance alone. Speed of germination is therefore a key phenotype of vigorous seeds and is known to differ with genetic background.

Mutations in many genes have been identified that show phenotypes with altered seed germination performance, and these have been instrumental in developing our current understanding of the control of germination (Finch‐Savage & Leubner‐Metzger, 2006; Weitbrecht *et al*., 2011; Graeber *et al*., 2012) and of seed vigour (Hilhorst & Toorop, 1997; Rajjou *et al*., 2012; Finch‐Savage & Bassel, 2016). However, the relative impact of these genes on wild‐type or crop seed performance is poorly understood and no clear candidates have been revealed that will form the basis of a discriminating test for seed vigour. Natural populations and contrasting crop genotypes offer an alternative source of genetic variation to laboratory‐induced mutations. Using this variation to identify QTL associated with seed vigour and the underlying genes influencing these traits provides a route to identify practically important genes.

In previous work we identified the principle QTL influencing Speed Of Germination (*SOG1*) located at the bottom telomeric end of chromosome 1 in the crop *B. oleracea* (Bettey *et al*., 2000). In the work presented here we use natural allelic variation in *B. oleracea* and exploit the resources available in this crop and the closely related model *Arabidopsis* to identify two genes within this QTL that negatively regulate speed of germination. We show that allelic variation in these *B. oleracea* genes alters germination speed and sensitivity of the phenotype to abscisic acid (ABA). We then further investigate the involvement of ABA and identify a second QTL (Reduced ABA 1 (*RABA1*)) responsible for differences in seed ABA content. We provide evidence that the responsible gene at this locus is a homologue of the ABA catabolic gene *AtCYP707A2*. We propose that collectively these three genes determine germination speed in *B. oleracea* by altering ABA content and sensitivity to it, and demonstrate that natural allelic variation in these genes could be harnessed to improve seed vigour.

## Materials and Methods

Molecular biological experiments and protocols, such as RNA extraction, PCR, Quantitative RT‐PCR, plant transformation, transgenic constructs, marker analysis, Illumina sequencing and data analysis were performed according to standard procedures detailed in Supporting Information Methods S1. Sample extraction and hormone analysis was performed using GCMS according to Finch‐Savage *et al*., 1992 or by UPLC/ESI‐MS/MS performed by the NRC Plant Biotechnology Institute, Saskatoon, Canada (Ross *et al*., 2004). All analyses were performed using the statistical package genstat 5 (Payne *et al*., 1993), and where appropriate data were transformed and subjected to analyses of variance.

### Plant and seed production and comparison of lines

*B. oleracea* **:** *SOG1* was identified using a doubled haploid mapping population derived from a cross between Chinese kale (var. *alboglabra*, A12DHd) and a Calabrese (var. *italica*, GDDH33) (Bohuon *et al*., 1996; Sebastian *et al*., 2000). Seed samples were obtained from Birmingham University, UK, for a number of chromosome substitution lines derived from the same parent lines (Rae *et al*., 1999). These lines (AGSL101, AGSL111, AGSL118 and AGSL119) each had introgressions of different sizes in the *SOG1* region from the GDDH33 parent in the background A12DHd parent. Bulk seeds were then produced from individual lines. Plants were laid out in a randomized block design in a glasshouse at 16–18°C during the 16 h day and at 10–15°C at night as described by Bettey *et al*. (2000). Supplementary lighting (400 W high pressure sodium lamps; Osram Ltd, St Helens, UK) was supplied when light intensity fell below 300 wm^2^ during the 16 h day. Plants were self‐pollinated by enclosing the inflorescences in perforated polyethylene bags containing blowflies before the flowers opened. The siliques were allowed to dry completely on the plant within the enclosing bags before harvest. The seeds were cleaned, equilibrated at 15% RH and 15°C, and then stored at −20°C before germination experiments were carried out.

In further experiments, BC_1_ seed from reciprocal backcrosses were produced in the same manner as described above. Bud pollination was performed to make the cross resulting in the BC_1_. The BC_1_ from backcrosses of AGSL101 onto A12DHd were then selfed and seeds produced. BC_1_S_2‐4_ plants were screened for presence of recombination using the markers described below. Lines identified with recombination in the AGSL101 introgressed region on chromosome 1 were selfed to BC_1_S_6_ under the production conditions described above. On all occasions seeds from the parent lines were produced at the same time for comparison to minimize the influence of environmental differences during seed production. Thus, comparisons between lines were always made on seed produced on the same occasion. Percentage germination was high (> 95%) in all seed lots.

Genomic DNA was extracted from young leaves of all plant material using a DNeasy plant mini kit (Qiagen).

*A. thaliana* **:** homozygous lines for *Arabidopsis* insertion mutants were selected by genomic PCR and sequence verification. Genomic DNA was extracted using CTAB extraction. Homozygous lines were selected for SALK lines using the LBb1.3 primer (ATTTTGCCGATTTCGGAAC) with the reverse primer (RP) as given at the SALK T‐DNA express website (http://signal.salk.edu/cgi-bin/tdnaexpress). Homozygous lines for the GABI KAT T‐DNA insertion line 579E09 inserted in At3g01090 (http://www.gabi-kat.de/) were determined using the T‐DNA primer (ATATTGACCATCATACTCATTGC) and a gene‐specific primer (TTGACCCATCAAATAATACACGAA). For the initial germination screen of all insertion mutant lines and wild‐type (WT) *COL‐0*, plants (six or greater) were grown under controlled environment conditions of 22°C during a 16 h day and at 18°C at night. Seeds were sown in *Arabidopsis* mix compost (6 : 1 : 1 Levingtons F1 compost : sand : vermiculite), in 5 × 5 × 5 cm P24 cells and individual plants were retained within aracons to prevent cross pollination. All lines including *COL‐0* bolted at the same time and set seed at the same rate. All seeds were harvested on the same day when siliques were air dry 3 wk after flowering. The speed of germination of freshly harvested seeds at 15°C was measured. The results from this screen indicated that four lines were faster germinating than the WT *COL‐0*. These lines were two independent T‐DNA insertions, verified by PCR, in each of the genes *At3g01060* (SALK_100860 and SALK_009486) and *At3g01150* (SALK_107494 and SALK_013673). To compare these four selected insertion mutant lines and *COL‐0*, 15 plants from each line were sown and raised as above, but in a randomized block design under glasshouse conditions as described above for *B. oleracea*. Seeds from individual plants were considered as biological replicates in the analysis.

In further experiments, seeds from *Arabidopsis* lines transformed with *B. oleracea* alleles were produced as described for insertion mutant lines. Mature air dry seeds from each plant were harvested 3 wk after the plant flowered to minimize differences in the period of after‐ripening on the plants. Speed of germination was compared in 24 independent WT plants, and 14 and 15 independent transgenic lines, containing A12DHd and GDDH33 alleles of *BoLCVIG1* (*At3g01060* homologue), respectively, which were considered as biological replicates in the analysis. In a separate experiment, speed of germination was also compared in 13 independent WT plants and 13 independent transgenic lines for A12DHd and for GDDH33 alleles of *BoLCVIG2* (*At3g01150* homologue), respectively, again considered as biological replicates in the analysis. In an experiment to investigate the impact of ABA, germination was compared between WT and five independent transgenic lines containing one or other of the two *B. oleracea* alleles of each gene. Seeds of the transgenic lines of each gene were produced at different times and so were tested separately; however, on each occasion seeds from WT control plants were produced for comparison.

Following harvest, all seeds were held at 55% RH for 2 d (termed freshly harvested) and then used immediately, stored at −80°C to minimize physiological change before germination experiments, or sealed and placed at 20°C to after‐ripen.

### Seed germination

In all germination experiments, seeds were placed on two layers of filter paper (Whatman International Ltd, Maidstone, UK) kept moist throughout with water (unless otherwise stated) in clear polystyrene boxes laid out in randomized blocks and kept either in the dark or light as described in the text. No evidence of fungal infection was observed and so seeds were not sterilized to avoid influencing their germination. Germination (radical emerged through the endosperm and seed coat) was recorded at frequent intervals to construct cumulative germination curves to allow an accurate calculation of the time to 50% germination (germination speed) from these measurements.

*B. oleracea* **:** Germination was recorded on three replicates of 50 seeds from all lines and treatments. Seeds from substitution and parent lines were placed to germinate on filter paper kept moist throughout treatment with water or solutions containing ABA (10, 33, 100 μM; Sigma‐Aldrich Co. Ltd, Dorset, UK), GA_4/7_ (10, 33 and 100 μM; Zeneca, Macclesfield, UK), a carotenoid biosynthesis inhibitor (1, 10, 38 μM Fluridone, Dow Chemical Co., Hitchin, UK) or GA synthesis inhibitors (100 and 500 μM; Prohexadione‐Ca, BASF Germany; 10 and 50 μM Paclobutrazole; Zeneca) at temperatures (± 0.5°C) described in the text. All methods and concentrations were studied in a series of preliminary experiments to determine effective treatments. All solutions were adjusted to a pH of 7. Fluridone, and GA synthesis inhibitors GA_4/7_ and GA_3_, were dissolved in ethanol initially and then diluted. The final concentration of ethanol was < 0.03%, which was found to have no significant influence on germination in preliminary studies. Controls were exposed to the same concentration of ethanol. In further experiments, seeds were placed to germinate at different water potentials on filter paper moistened with polyethylene glycol (PEG 8000; BDH Ltd, Poole, UK) solutions. Water potentials were set by the concentration of PEG (Bradford, 1995; Finch‐Savage, 2004). The same solution volume : filter paper ratio was used for germination as that in the vapour pressure osmometer (model 5100C; Wescor Inc., South Logan, UT, USA) used to measure water potential, thus avoiding the potential error resulting from filter paper exclusion of PEG. Hydrothermal time analysis was performed according to Finch‐Savage (2004).

As part of a larger unpublished comparison of seedling emergence from *B. oleracea* genotypes, 100 seeds of GDDH33, A12DHd and AGSL101 were sown on 31 May 2003, in four replicate 1‐m rows arranged in a randomized block design. Seeds were sown by hand in a 15‐mm‐deep furrow, covered with sieved soil (sieve hole size < 4 mm) and the surface rolled once with a Stanhay seed drill press wheel. The soil was a sandy loam and irrigation was applied to maintain soil moisture throughout seed germination and seedling emergence. The latter was recorded at regular intervals until no more seedlings emerged.

*A. thaliana*: Where stated in the text, seeds were placed to germinate immediately following harvest (freshly harvested), following after‐ripening or after cold treatment at 5°C for 3 d. Germination was recorded on 40–50 seeds from each individual plant regarded as biological replicates.

### Seed development and imbibition samples

*B. oleracea* **:** Seed samples were collected at six times from flowering to seed maturity; and a further six time points during seed imbibition to germination completion (radicle emergence). For seed development plants were produced in a glasshouse as described above in four replicate blocks. Plants were allowed to produce flowers for 14 d and then all further flowers were removed to limit the spread in development between seeds. At each harvest point, two plants were taken from each replicate block. Seeds were extracted and combined to provide a stock of four replicate seed bulks. For imbibition samples, four replicates of *c*. 250 seeds for each sample time were placed to imbibe on moistened paper at 15°C in the light. At appropriate times they were removed and blotted dry. Seed samples taken during development and imbibition were flash frozen in liquid nitrogen and stored at −80°C until RNA extraction.

## Results

### *SOG1* locus on *B. oleracea* chromosome 1 confirmed and characterized

**Figure 1:**
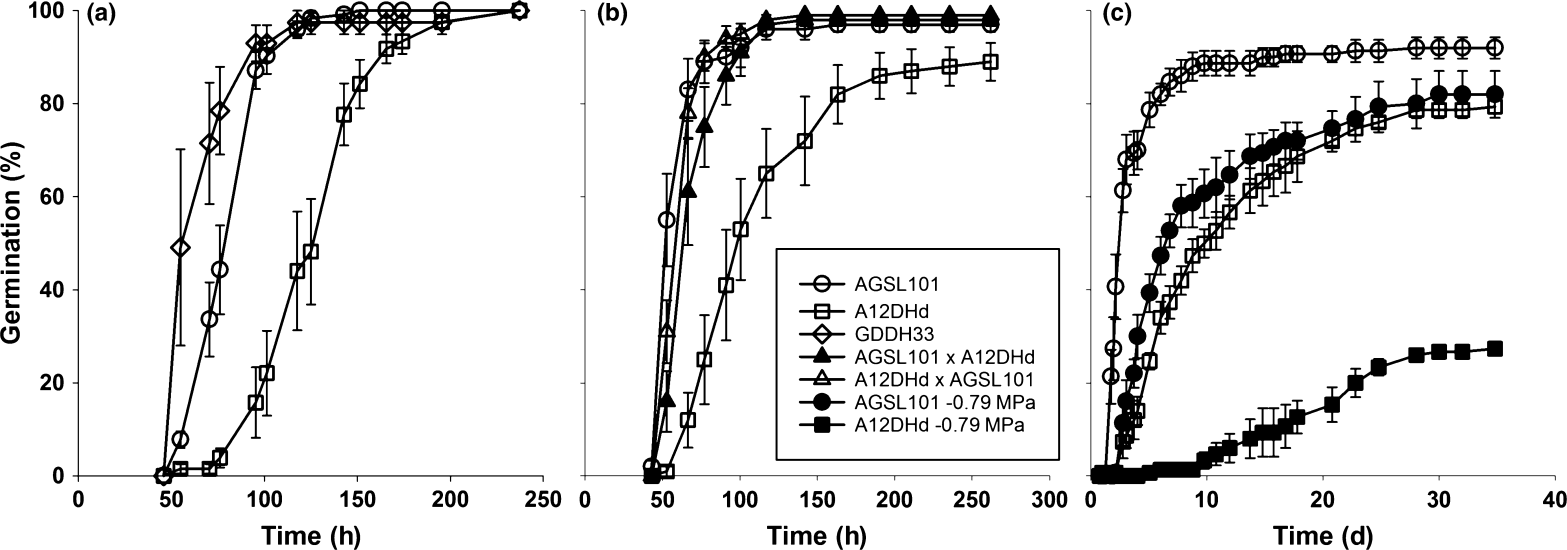
Cumulative *Brassica oleracea* seed germination curves. (a) Comparison of the substitution line AGSL101 and parent lines (A12DHd and GDDH33) on water. (b) Comparison of AGSL101, the recurrent A12DHd parent and reciprocal BC_1_ backcross lines (A12DHd × AGSL101 and AGSL101 × A12DHd). (c) The influence of water potential (0 and −0.79 MPa) on the germination of seed from AGSL101 and A12DHd. All germination was carried out on three replicates of 50 seeds at 15°C in the light. Error bars are ± SE.

Speed of germination (*SOG*) was compared between *B. oleracea* doubled haploid parent lines A12DHd (var. *alboglabra*) and GDDH33 (var. *italica*). ANOVA of germination data showed that the GDDH33 parent germinated significantly (*P *<* *0.001) faster (lower T50, time to 50% germination) than the A12DHd parent (Fig. 1a). This confirms that the positive germination rate alleles are provided by GDDH33 as shown by Bettey *et al*. (2000).

Analysis of informative chromosome substitution lines (Rae *et al*., 1999) derived from these parent genotypes identified four substitution lines each having GDDH33 introgressions in the A12DHd background that spanned the *SOG1* QTL (AGSL101, AGSL111, AGSL118, AGSL119). In replicate experiments, these lines all had significantly (*P *<* *0.05) lower T50 than the A12DHd parent, indicating faster germination (Figs 1a, S1a). This result was confirmed in a repeat of the experiment and there was no difference found in the percentage germination between lines on either occasion. Of the four substitution lines, AGSL101 has the smallest introgressed region at *SOG1* (1–9 cM; Rae *et al*., 1999), it accounted for much of the difference in germination rate between the parent lines, and it tended to germinate faster than the other three substitution lines (Figs 1a, S1a). AGSL101 was therefore selected for further analysis and fine mapping described below.

### Speed of germination is determined by the embryo

Reciprocal backcrosses were made between AGSL101 (GD33 alleles at *SOG1*) and A12DHd so that the BC_1_ seeds produced would have embryos that are heterozygous and contain alleles from both parents in the *SOG1* region. However, the surrounding maternal tissues would be either AGSL101 or A12DHd, and seed performance would therefore differ if there were a maternal influence. There was no significant difference in T50 between AGSL101 and the BC_1_ from the reciprocal backcrosses with A12DHd, but the T50 of seeds from all three was significantly (*P* < 0.01) less than that from seeds of A12DHd (Fig. 1b). The faster germinating GDDH33 alleles are therefore dominant. Furthermore, no genetic maternal influence on inheritance of the trait was found, indicating it to be embryo‐based. This result was confirmed by comparison of the seeds from GDDH33 and A12DHd and their reciprocal backcrosses.

### Beneficial alleles at *SOG1* in AGSL101 confer enhanced stress tolerance in addition to more rapid germination

Seedling emergence from AGSL101 and the parent lines was compared following sowing the seeds in moist sandy loam soil under field conditions. Seedling emergence involves both germination and subsequent downward radicle growth and upward hypocotyl extension growth to carry the cotyledons above the soil surface. Under these low‐stress conditions, seedlings from AGSL101 and GDDH33 had a time to 50% emergence of 10.9 and 11.1 d, respectively, which is significantly (*P *<* *0.05) earlier than 12.0 d from A12DHd seeds. Under suboptimal field conditions where seed/seedlings experience multiple stresses (Whalley & Finch‐Savage, 2006), the difference between seedling emergence times from these lines is likely to be enhanced (Finch‐Savage & Bassel, 2016). In the laboratory, even under relatively mild water stress (−0.79 MPa), the more rapid germination from AGSL101 compared to that of A12DHd was greatly exaggerated (Fig. 1c). This affect was modelled using hydrothermal time (HTT; Bradford, 1995; Fig. S1B). Within the model, Ψ_*b*_ (base water potential) is a measure of the sensitivity of germination to decreased water potential. The more negative Ψ_*b*_(50) (Ψ_*b*_ of the 50^th^ percentile) calculated for AGSL101 (−1.2 MPa) and GDDH33 (−1.5 MPa) than that of A12DHd (−0.6 MPa) generalizes their greater tolerance to water stress and their greater speed of germination.

### Identification of genetic markers across *SOG1* in *B. oleracea* to facilitate fine mapping

There is a close genetic relationship between the model *Arabidopsis* and the crop *B. oleracea*. Previously, synteny has been shown between a number of regions in the *Brassica* C genome and *Arabidopsis* (Cogan *et al*., 2004; Parkin *et al*., 2005). At the start of this work, a region at the bottom telomeric end of *B. oleracea* chromosome 1 that contains *SOG1* was identified as having possible synteny with the upper arm of *Arabidopsis* chromosome 3. To confirm this synteny, we developed gene markers specific to *SOG1*. These markers were used to screen two *Brassica oleracea* Bacterial Artificial Chromosome (BAC) libraries (JBo and BoB; www.brassica.info/clones) to select BACs for the construction of a tiling path that spanned the introgressed region in AGSL101, which defined *SOG1*. From this we identified five markers (Marker 1 = *At3g03110*; Marker 2 = *At3g02920*; Marker 3 = *At3g02555*; Marker 4 = *At3g02090*; Marker 5 = *At3g01150*) spread across the region that were polymorphic between A12DHd and GDDH33 parent alleles for use in the genetic screening outlined below. This process is summarized in Fig. S2.

### A backcross inbred line (BIL) population was produced and subjected to genetic and phenotype screening to fine map *SOG1* in *B. oleracea*

AGSL101 containing the introgression defining *SOG1* was backcrossed to the slow germinating background A12DHd parent to create a population of backcross lines. The population of > 1300 lines (BC_1_F_2–4_ for A12DHd × AGSL101) was screened with the five polymorphic markers described above to identify lines with recombination break points in *SOG1*. These lines were identified and selfed to the F_6_ generation to create a unique set of Backcross Inbred Lines (BILs) to fine‐map *SOG1* (Fig. S3a). These BILs were screened for germination at the low temperature of 8°C to slow germination and allow more recordings and thus more accurate cumulative germination curves to determine speed of germination. These data were used to refine *SOG1* through statistical analysis of the genotype scores (Fig. S3a,b). This process fine‐mapped *SOG1* to a region defined by two of the markers (Marker 4, *At3g02090* and Marker 5, *At3g01150*). Furthermore, the analysis showed speed of germination was significantly different at markers 4 and 5. Thus, genes associated with both markers independently influence *SOG1*. We then extended the statistical analysis to look for linkage at pairs of markers and found that markers 4 and 5 were significantly linked (*P *<* *0.012), suggesting that genes associated with these markers influence speed of germination in the same way. The refined QTL was confirmed using germination data from the F_5_ generation.

### A single BAC contains *SOG1*

One of the BACs (BoB064L23) in the tiling path constructed over *SOG1* (Fig. S2) contained both molecular markers 4 and 5, which defined the fine‐mapped *SOG1* locus. A full annotation of this BAC is deposited in GenBank AC KM027339. Sequencing revealed the BAC contains 12 full‐length genes (Table S1), a number of truncated genes and two regions of retro elements. BoB064L23 covers sequence at the bottom telomeric end of the chromosome and we have been unable to identify further sequence distal to this BAC. We therefore considered that our candidate genes were among the 12 identified.

### Analysis of *Arabidopsis* insertion mutant lines and *B. oleracea* BILS identify two linked genes that independently influence germination in *SOG1*

**Figure 2:**
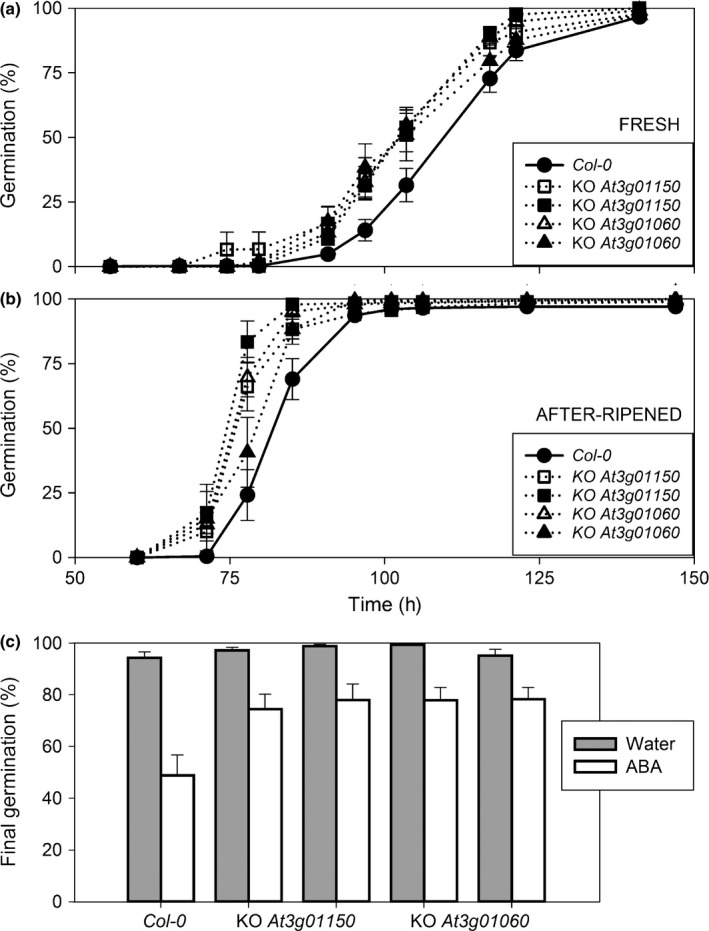
*Arabidopsis* insertion mutants at *At3g01150* and *At3g01060* germinate faster than wild‐type (WT) *COL‐0*. (a) Cumulative germination curves of freshly harvested seeds from WT *COL‐0* and two independent T‐DNA insertion lines in each of *At3g01150* and *At3g01060*. (b) Cumulative germination curves following dry after‐ripening (equilibrium with 55% RH for 1 month). (c) Final percentage germination on 1 μM abscisic acid (ABA). Germination was recorded on 15 replicates of 40 seeds at 15°C in the light. Error bars are ± SE.

We obtained all available SALK and GABI KAT T‐DNA insertion mutant lines in *Arabidopsis* for the genes on BoB064L23 (seven of the 12 genes present; Table S2a). Analysis of these lines revealed that only two of these genes (*At3g01060*,* At3g01150*) significantly influenced the germination phenotype in *Arabidopsis* (Fig. 2). We had two independent lines with different T‐DNA insertions in each of these two genes (Table S2b). The insertion lines all had significantly (*P* < 0.05) lower T50 than *COL‐0*, but final percentage germination was not affected (Fig. 2a). The same fast‐germinating phenotype from two independent insertion events in each gene is strong evidence that they both determine the germination influence of the *SOG1* QTL. An insertion in either of these genes increased germination speed, indicating that both genes have a negative effect on speed of germination.

Further characterization showed that following dry after‐ripening (to remove any residual dormancy) the insertion lines remained significantly (*P *<* *0.05) faster germinating than the WT (Fig. 2b), and they were significantly (*P *<* *0.001) less sensitive to exogenous ABA (1 μM) than the WT (Fig. 2c). Endogenous [ABA] was measured and was not significantly different between the WT and mutant lines (*COL‐0 *=* *181 ng g^−1^ DW, SD* *=* *41; *At3g01150* mutant* *=* *168 ng g^−1^ DW, SD* *=* *44; *At3g01060* mutant* *=* *207 ng g^−1^ DW, SD* *=* *59).

According to nomenclature agreed by the *Brassica* research community (Østergaard & King, 2008), *B. oleracea* homologues of *At3g01060* and *At3g01150* were named *BoLCVIG1* (*Bo1g157540*) and *BoLCVIG2* (a; *Bo1g157630* and *b; Bo1g157580* for the two copies in *SOG1;* Table S1), respectively. Both copies of *BoLCVIG2* in *SOG1* have a high degree of sequence similarity (> 90%) with *At3g01150*. For use here and throughout this study, we produced RNAseq data on seed development and imbibition samples (each were combined samples from six time points) for both AGSL101 and A12DHd genotypes. Using these data we found no difference in expression of either copy of *BoLCVIG2* between the genotypes during seed development or imbibition. However, compared to *BoLCVIG2a*, expression of *BoLCVIG2b* was minimal; thus, further work focused on *BoLCVIG2a*, henceforth called *BoLCVIG2*. Out of the 12 full‐length genes identified on BoB064L23, only *BoLCVIG1* showed a significant difference (described below) in gene expression between the genotypes.

Further marker development on the BILs subsequently showed that *BoLCVIG1* and *BoLCVIG2* were grouped with markers 4 and 5, respectively, and that a subset of the BILs contained a recombination event between them (Fig. S3). Thus, evidence is provided in both *Arabidopsis* (insertion mutant lines) and *B. oleracea* (gene expression; fine‐mapping analysis of BILs) that there are two linked genes which independently determine germination speed in *SOG1*.

### Transformation of *Arabidopsis* with natural variants of *B. oleracea* homologues of *At3g01060* and *At3g01150* (*BoLCVIG1* and *BoLCVIG2*) confirm their influence on germination speed

Our work above shows that the *SOG1* region in *B. oleracea* shared synteny with the upper arm of *Arabidopsis* chromosome 3. This region in *Arabidopsis* also contains collocated QTL for germination speed and germination tolerance to ABA and the stresses of controlled deterioration and salt (Clerkx *et al*., 2004). Clerkx *et al*. (2004) show that the *Arabidopsis* accession *Sha*, used in this work, is fast germinating and one of the most tolerant accessions to various seed stresses. In their QTL analysis *Sha* was found to have positive alleles (fast germination) compared to the other accession for all the traits mapped to this locus (Clerkx *et al*., 2004). Thus, we used the *Sha* accession as a stringent test for these genes that we show above have a negative effect on speed of germination (Fig. 2). We therefore tested the influence of natural homologous *B. oleracea* alleles of these genes by cloning the parental *B. oleracea* alleles (A12DHd and GDDH33) of both genes (*BoLCVIG1* and *BoLCVIG2*) including their native promoters and transformed them into *Arabidopsis* (*Sha*).

**Figure 3:**
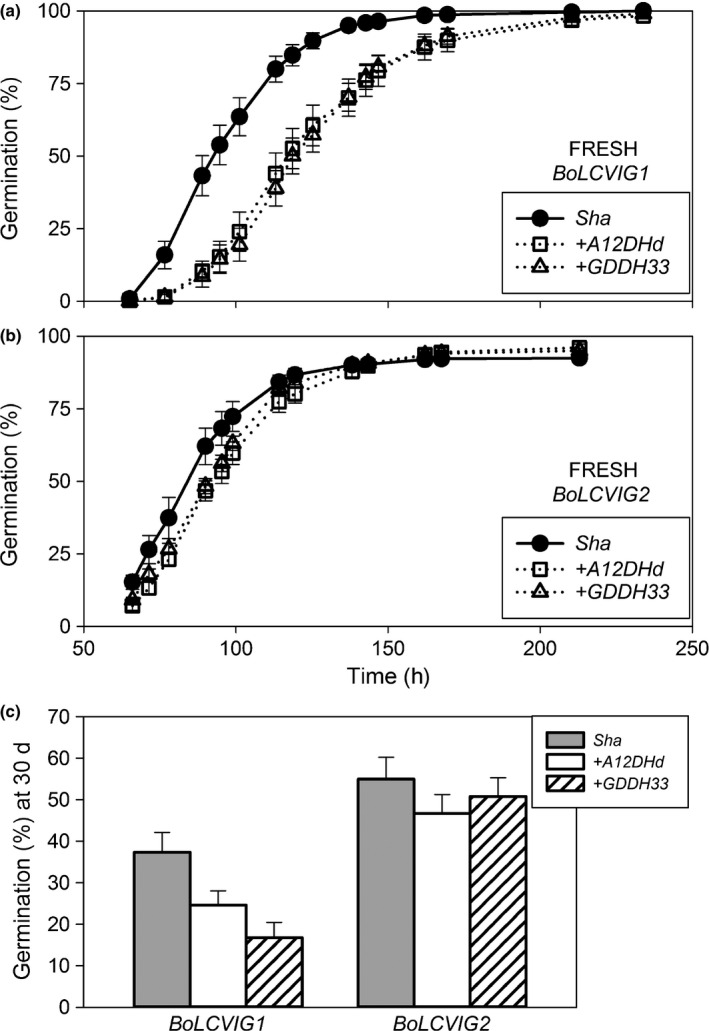
Freshly harvested seeds of *Arabidopsis* (*Sha*) transformed with *BoLCVIG1* or *BoLCVIG2* alleles from both parents (A12DHd and GDDH33) germinate more slowly than wild‐type (WT) *Sha* seeds on water. Cumulative germination curves are shown for seeds transformed with (a) *BoLCVIG1 and* (b) *BoLCVIG2*. (c) Percentage germination at 30 d after sowing on 2.25 μM abscisic acid (ABA). Germination was recorded at 15°C in the light. Seeds of lines transformed with alleles of *BoLCVIG1* or *BoLCVIG2* were produced at different times and on each occasion seeds from WT *Sha* control plants were produced. Data are the means of at least five independent transformations as specified in the Materials and Methods section, and presented alongside the appropriate control for comparison. Each transformation was represented by a replicate of 50 seeds. Error bars are ± SE.

T‐DNA insertions in *At3g01060* and *At3g01150* in the *Col‐0* background increased germination speed and enhanced ABA tolerance. However, freshly harvested seeds of transgenic lines of both alleles of *BoLCVIG1* showed the opposite response and germinated significantly (*P *<* *0.001) slower (higher T50) than the *Sha* WT seed (Fig. 3a), thus confirming the effect of these alleles on speed of germination. However, there was no significant difference in the speed of germination between transformed lines containing the different *B. oleracea* alleles. In a separate experiment, transgenic lines containing *BoLCVIG2* also tended to germinate slower than the WT *Sha* seed, but the differences between alleles were not significant (Fig. 3b).

From preliminary experiments, 2.25 μM was selected as the most informative ABA concentration. This ABA concentration reduced final percentage germination to *c*. 60% and 80% in seeds from the separate production of lines transformed with *BoLCVIG1* and *BoLCVIG2*, respectively. However, it did not significantly alter final percentage germination between the lines and their WT *Sha* controls produced at the same time. Speed of germination was not significantly different between lines transformed with *BoLCVIG2* alleles and their WT control, but lines transformed with *BoLCVIG1* alleles were slower germinating than their WT control. Thus, at 30 d the percentage germination of lines transformed with alleles of *BoLCVIG1* were significantly (*P *<* *0.001) less than their WT control (Fig. 3c). This experiment was repeated and gave the same result. On both occasions, ABA solutions were changed regularly and so the result was not influenced by photodegradation of ABA.

### *BoLCVIG1* is a single copy gene transcribed in different alternatively spliced isoforms

**Figure 4:**
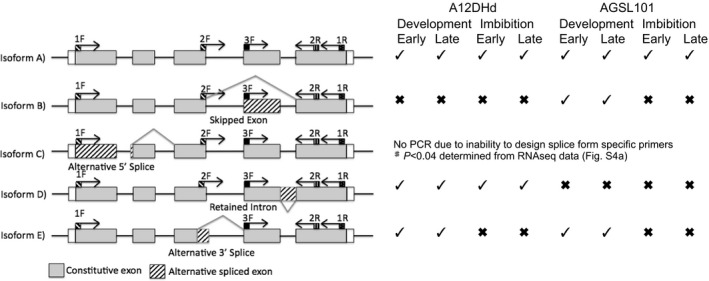
A schematic illustration of five isoforms (A–E) of *BoLCVIG1* and their expression during early and late seed development and imbibition. The presence (tick) or absence (cross) by PCR of isoforms A, B, D and E at the different stages of development and imbibition are shown. PCR was not possible for isoform C, but a significant (♯) difference between genotypes across development and imbibition was determined from RNAseq data (Supporting Information Fig. S4a). In the schematic: primers used for PCR amplification of the transcripts are indicated by arrows; boxes on the solid black line indicate exons; white boxes indicate 3^′^ and 5^′^ untranslated regions; and boxes with diagonal stripes indicate a deletion.

Genomic and RNAseq data revealed there was a single copy of *BoLCVIG1* in both genotypes that was expressed during both seed development and imbibition, but that expression in both stages was more than two‐fold higher in A12DHd than AGSL101 (Fig. S4a). Verification of its unique nature was obtained through BLASTn comparison with the TO1000DH genome sequence (Parkin *et al*., 2014). We found no evidence that either copy of *BoLCVIG2* was spliced alternatively. However, RNAseq data indicated there were different isoforms of *BoLCVIG1* and this was confirmed using PCR (Figs 4, S4a). Although there was at least one isoform present at both early and late stages of seed development and imbibition, there were isoforms that were stage‐ (isoforms B, C, E) and genotype‐specific (isoforms B, C, D). Notably isoform D was present in A12DHd during seed development and imbibition, but was not found in AGSL101. The expression of isoform C was significantly (*P* < 0.04) different between genotypes (Fig. 4). There is evidence that regulation of splicing differs between *Arabidopsis* and other *Brassicaceae* (Nellist *et al*., 2014) and this may explain the limited difference in phenotype between *Arabidopsis* lines transformed with different *B. oleracea* alleles (Fig. 3a,b).

### Allelic variation at a further QTL (*RABA1*) determines seed ABA content in *B. oleracea*

We investigated ABA content as the cause of differences in seed vigour resulting from *SOG1*. ABA content was measured in the seeds of BILs and found to differ greatly between lines (up to 10‐fold; < 200 > 1400 ng g^−1^ DW) and to significantly correlate (*R *=* *0.411, df 33; *P *<* *0.025) with their T50 (Fig. S5a, b). However, BILs could be grouped into two clear categories, higher and lower ABA content (> or < 300 ng ABA g^−1^; Fig. S5b), but this grouping could not be explained by the recombination in the *SOG1* locus of the BILs, suggesting that another factor was involved.

**Figure 5:**
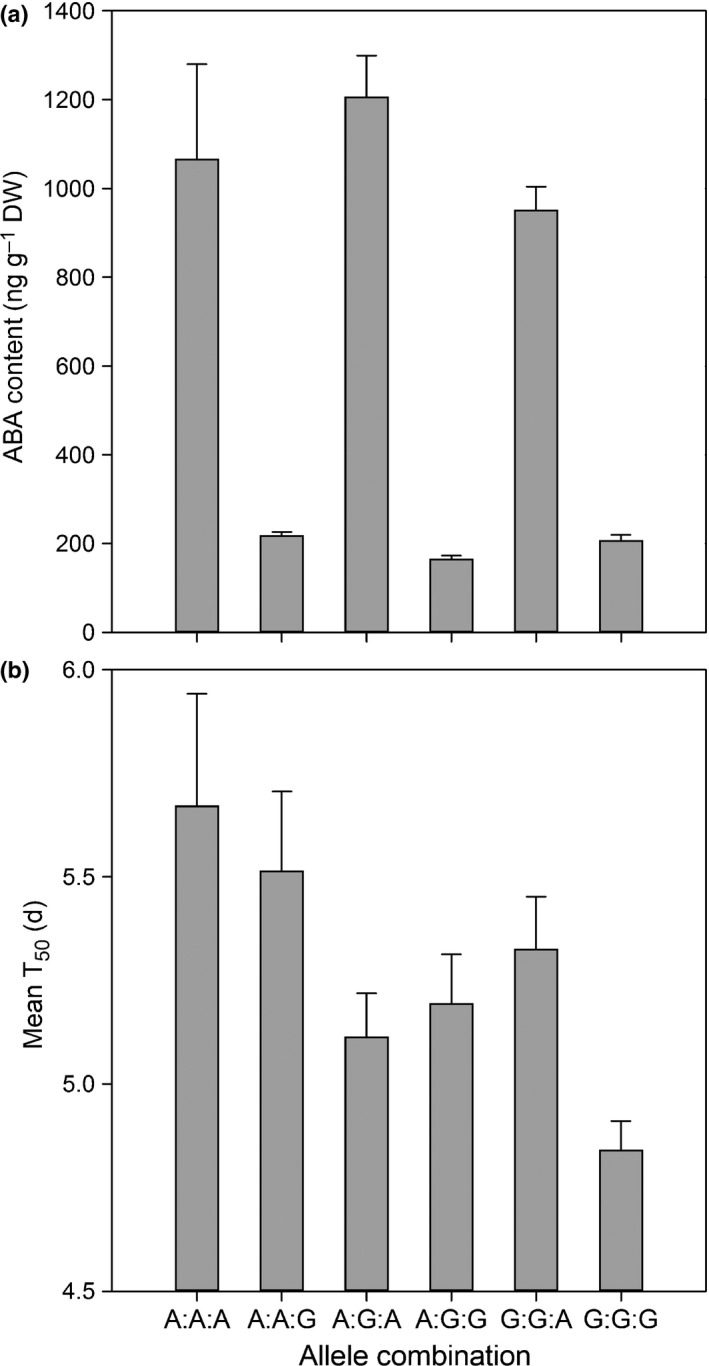
Abscisic acid (ABA) and speed of germination (T50) in *Brassica oleracea* BILs. (a) The mean (a) ABA content and (b) T50 at maturity are shown on the *y*‐axes for BILs with different combinations of alleles either A (A12DHd) or G (GDDH33) at three loci (*BoLCVIG1: BoLCVIG2*:*RABA1*) shown on the x axis (e.g. A : A : A, A : A : G etc.). Germination was recorded on three replicates of 50 seeds at 15°C in the light. Error bars are ± SE.

*SOG1* was reported to be the only introgression in AGSL101 (Rae *et al*., 1999). However, we tested this assumption by challenging AGSL101 with 1108 polymorphic KASP^™^ SNP markers and we found three introgressions. Re‐genotyping the BILs with these same markers confirmed recombination in chromosome 1, but also found recombination in introgressions on chromosomes 4 and 8. Comparison of markers with the ABA phenotype data revealed an exact correlation with high ABA content assigned to the A12DHd genotype and low ABA content assigned to the GDDH33 genotype at four markers in the recombined region on chromosome 4 (Fig. S5b). These four markers therefore define a new locus *RABA1* (Reduced ABA 1) influencing ABA content in *B. oleracea* seeds. The allele (A12DHd or GDDH33) present at this locus has a highly significant (*P *<* *0.001) effect on the ABA content of that line (Figs 5a, S5b). Nevertheless, analysis of the A12DHd × GDDH33 population genotyped with the same KASP^™^ SNP markers and the original speed of germination screen data that identified *SOG* QTL (Bettey *et al*., 2000), confirmed that there was no significant or indicative *SOG* QTL on chromosome 4 where *RABA1* was located.

### Allelic variation in *SOG1* affects speed of germination and alters sensitivity to ABA in *B. oleracea*

Further analysis of the BILs is provided in Fig. 5 for ABA content and the mean speed of germination (T50) of lines with different allele combinations (A12DHd (A) or GDDH33 (G)) at the three loci (*BoLCVIG1*:* BoLCVIG2*:* RABA1*). The figure shows that the speed of germination and the influence of ABA content differed depending on the combination of alleles present at these loci.

ABA content was significantly (*P* < 0.001) higher when the A12DHd allele (A) was present at the *RABA1* locus compared to when the GDDH33 allele (G) was present (Fig. 5a). However, this did not have a consistent effect on speed of germination as the effect of ABA content on this phenotype depended on the alleles present at the two genes in *SOG1* (Fig. 5b). When there was an A12DHd allele present at both *BoLCVIG1* and *BoLCVIG2*, germination was slow and unaffected by the allele at *RABA1*. When there was a GDDH33 allele present at *BoLCVIG2*, germination was faster but also unaffected by the allele at *RABA1*. However, when there was a GDDH33 allele present at *BoLCVIG1,* germination was significantly (*P *<* *0.01) faster when there was also a GDDH33 allele at *RABA1* and therefore ABA content was low.

Thus, the alleles at all three loci influence speed of germination; the allele at *RABA1* determines ABA content, that at *BoLCVIG2* modulates speed of germination and that at *BoLCVIG1* affects sensitivity of this phenotype to the ABA content. The GDDH33 alleles present in AGSL101 (i.e. G : G : G) are all beneficial in terms of seed vigour compared to those from the slow‐germinating A12DHd parent (i.e. A : A : A).

### The activity of a homologue of *Arabidopsis CYP707A2* present at *RABA1* is consistent with differences in ABA content

The four markers described above as defining the *RABA1* locus (Fig. S5b) cover a section of *B. oleracea* chromosome 4 between the genes *Bo4g165220* and *Bo4g169329* as described in the TO1000 genome assembly of *B. oleracea* numbered GenBank: JJMF00000000.1. This region contains 114 genes including transposable elements and hypothetical proteins. To reduce the list of candidate genes responsible for the recorded difference in ABA content between AGSL101 and A12DHd due to *RABA1*, we compared expression of all the genes in the region in both genotypes using the RNAseq data previously described (combined samples collected across six time points during seed development and during imbibition). We found only three genes during seed development and two during imbibition, in which expression differed significantly between these two genotypes (Fig. S5c). Only one of these genes, *Bo4g168080*, was found to be common to both samples, showing either a 46.5‐fold (development) or a 3.8‐fold (imbibition) upregulation in AGSL101 compared to A12DHd. Bioinformatic analysis found that this gene sequence shared a 95% similarity with *CYP707A2* from the *Brassicaceae*,* Sisymbrium officinale* and 89% similarity in *Arabidopsis*. *AtCYP707A2* is a key ABA catabolic gene identified in *Arabidopsis* seeds and the *cyp707a2* mutant has greatly increased ABA content in its seeds, which consequently exhibited hyperdormancy compared to those of the WT (Kushiro *et al*., 2004).

**Figure 6:**
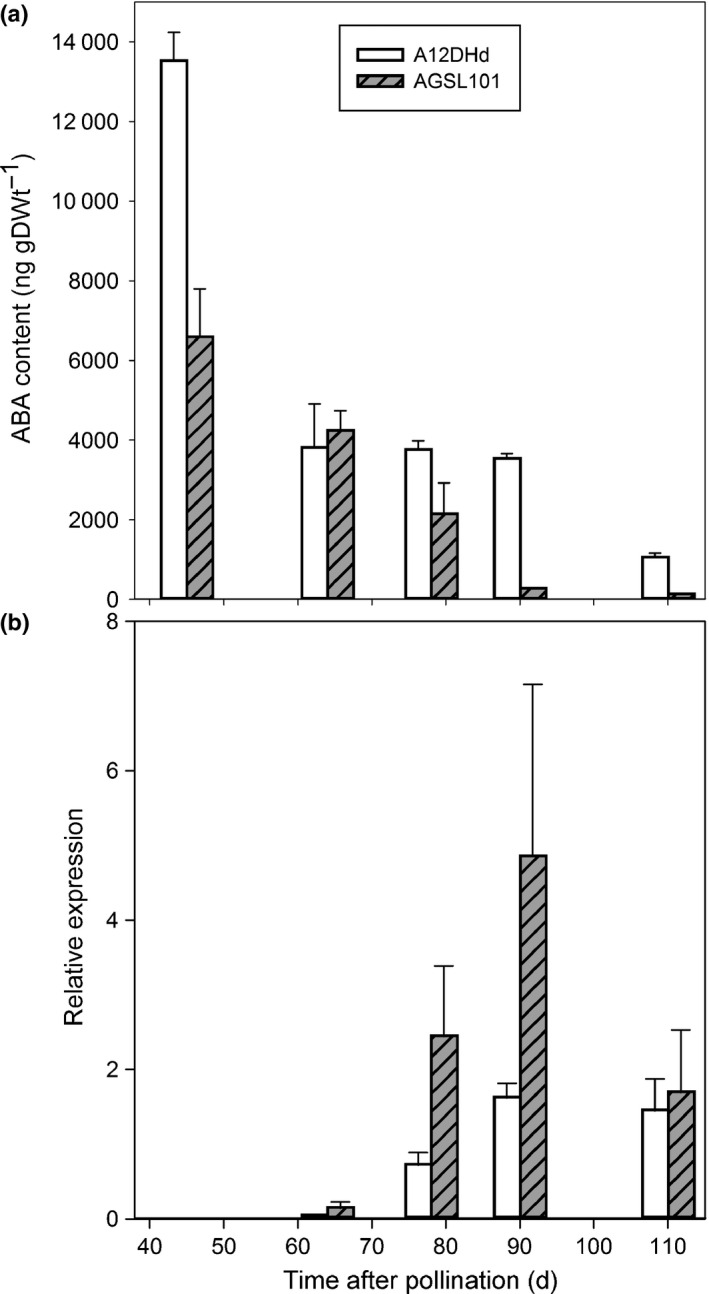
Abscisic acid (ABA) content and *BoCYP707A2* expression in *Brassica oleracea*. (a) Endogenous concentrations of ABA and (b) expression of *BoCYP707A2* for AGSL101 and A12DHd at intervals during seed development in *B. oleracea*. Error bars are ± SE based on two and three replicates for ABA and expression measurements, respectively.

In order to investigate further, we measured ABA content (Fig. 6a) and used quantitative PCR to follow the expression of *BoCYP707A2* (Fig. 6b) at intervals during seed development. ABA content is initially higher in A12DHd than AGSL101, but this difference is lost during subsequent decline in both genotypes. *BoCYP707A2* expression was found following 60 d after pollination (DAP) and ABA continued to decline in AGSL101 to a greater extent than in A12DHd consistent with higher expression of *BoCYP707A2* in the former. This is consistent with the subsequent difference in ABA content at maturity where lines having A12DHd alleles at *RABA1* have higher ABA content than those with GDDH33 alleles (Fig. 5a). We therefore suggest that the behaviour of *BoCYP707A2* is consistent with being responsible for the ABA phenotypes resulting from the allelic variation between AGSL101 and A12DHd present at the *RABA1* locus.

We used the carotenoid and ABA synthesis inhibitor Fluridone to determine whether there was ABA synthesis during imbibition. Fluridone applied at a range of concentrations had no significant effect on speed of germination or percentage germination suggesting that there is no active synthesis of ABA during imbibition under optimum conditions. Therefore, differences in endogenous ABA concentrations resulted from synthesis and catabolism during seed development only. Emerging cotyledons from Fluridone‐treated seeds were chlorotic, providing visual evidence that the treatment had been effective (i.e. inhibited carotenoid synthesis). Exogenous application of ABA delayed time to germination in AGSL101 and both of its parent lines (A12DHd and GDDH33); however, the extent of reduction in T50 by ABA differed significantly (*P* < 0.05) between these genotypes and was greatest in seeds of A12DHd.

## Discussion

### Characterization of *SOG1* and *RABA1*

Quantitative trait loci (QTL) for many seed performance‐related traits in *Arabidopsis* (Clerkx *et al*., 2004; Bentsink *et al*., 2010; Joosen *et al*., 2012; Nguyen *et al*., 2012) collocate at the top of *Arabidopsis* chromosome 3. These publications show that the region has an important role in many aspects of seed performance including germination speed, stress resistance, depth of dormancy and longevity. The chromosomal region of *Brassica oleracea* that contains the QTL *SOG1* was shown both here and elsewhere (Parkin *et al*., 2014) to be collinear with this section of the *Arabidopsis* genome. Using the *B. oleracea* substitution line AGSL101, we isolated *SOG1* of the faster‐germinating GDDH33 parent in the genetic background of the recurrent slow‐germinating parent A12DHd. The locus was dominant and accounted for much of the difference in speed of germination between the parents and the relative difference was retained following after‐ripening. Germination speed was unaffected by the maternal parent and therefore not influenced by the seed coat.

Hydrothermal time analysis demonstrated that AGSL101, which had GDDH33 alleles at *SOG1* in an A12DHd background, had seeds that were more environmentally robust than the A12DHd parent. The more rapid germination of AGSL101 and GDDH33 seeds resulted from lower threshold (base) water potentials for germination than that in seeds of A12DHd. This lower threshold will result in a predictably greater tolerance to water stress and indicates lower sensitivity to the germination inhibitor abscisic acid (ABA) (Ni & Bradford, 1992). We found also that there were differences in ABA content in the informative Backcross Inbred Lines (BILs) selected from the AGSL101 onto A12DHd backcross population and this was determined by alleles present at the *RABA1* locus on *B. oleracea* chromosome 4.

### The impact of allelic variation at *SOG1* and *RABA1* on seed vigour

ABA content in the seeds of the BILs (AGSL101 backcrossed to A12DHd) differed by up to 10‐fold. Across these lines there was a significant (*P *<* *0.05) positive relationship between their ABA content and T50. However, this overall correlation masked an important finding that the allelic form of genes at the *SOG1* locus determined this relationship. The allele present at *BoLCVIG2* altered the speed of germination (lower T50 when GDDH33 allele present); and when the GDDH33 allele was present at *BoLCVIG1* T50 was lower when ABA content was lower, indicating a difference in sensitivity to ABA. The influence of *BoLCVIG1* on the impact of *RABA1*‐determined differences in ABA content is consistent with *RABA1* not being identified as a *SOG* QTL.

These data erroneously suggest that lines with A12DHd alleles at *SOG1* were not ABA sensitive, but this is incorrect because exogenous application of ABA has a greater negative effect on the germination of A12DHd seeds than AGSL101 seeds. We therefore suggest that those BILs with A12DHd alleles in *SOG1* are highly sensitive and therefore negatively affected at low ABA contents, and do not respond further to an increase in ABA content. By contrast, those BILs with GDDH33 alleles at *BoLCVIG1* are only influenced at the very high contents present in lines with A12DHd alleles at *RABA1*. Such differences in sensitivity may help to explain why there are different relationships between ABA content and dormancy or seed vigour characteristics reported in the literature. This result is also in line with literature showing that ABA is essential, but does not determine the depth of seed dormancy (Footitt *et al*., 2011; Nakabayashi *et al*., 2012).

In crops, the seed should ideally be sensitive to ABA during development to prevent pre‐harvest sprouting, but unlike wild plants, be insensitive to ABA during imbibition to facilitate rapid germination (high vigour). This situation is not generally present in nature because it does not bestow an evolutionary advantage and seeds are more normally dormant at shedding (Finch‐Savage & Basal, 2016). This trait therefore appears to be a product of domestication, which may explain the greater influence of this QTL here in the crop *B. oleracea* than in the model *Arabidopsis* that has not undergone domestication. The influence of *BoLCVIG1* at these stages presents a mechanism for this with potential for optimization to enhance seed vigour in crops. Initial studies indicate that beneficial alleles of these genes can be introduced to enhance *B. oleracea* seed performance (Finch‐Savage *et al*., 2013) in commercial practice.

### Genes underlying *SOG1* and *RABA1*

We identified two genes at *SOG1, BoLCVIG1* and *BoLCVIG2,* which gave a slower germinating phenotype when alleles from both *B. oleracea* parents were introduced into Arabidopsis. The genes are homologues of the *Arabidopsis* genes *At3g01060* and *At3g01150*, respectively. In *Arabidopsis* both genes have been associated with Phytochrome Interacting Factor (PIF) family members that are important in the regulation of seed dormancy and germination by environmental cues including light and temperature (Penfield *et al*., 2010). In *Arabidopsis*,* At3g01150* was found to be a homologue of a specific vertebrate polypyrimidine tract‐binding protein (Wang & Brendel, 2006) and was termed AtPTB1 (Wang & Okamoto, 2009). This gene along with two other homologues (AtPTB2 and AtPTB3) can regulate alternative splicing (Stauffer *et al*., 2010). More specifically, AtPTB1 and 2 showed redundancy between them and both were shown to regulate alternative splicing of *PHYTOCHROME INTERACTING FACTOR6* (PIF6) coinciding with reduced primary dormancy (Penfield *et al*., 2010) and altered rates of ABA‐dependent seed germination (Ruhl *et al*., 2012). *PTB1* appears to impact on many genes (Stauffer *et al*., 2010; Ruhl *et al*., 2012) and so may have a much wider role because splice variants are being found in an increasing number of genes (Syed *et al*., 2012) including key regulators of germination such as *DOG1* (Bentsink *et al*., 2006). In RNAseq data we found no evidence for a difference in *BoDOG1* or *BoCYP707A2* isoforms between the genotypes studied. However, for both copies of *BoPIF6* present within the *B. oleracea* genome the same two isoforms were found (Fig. S4B), but these isoforms differed from those identified in *Arabidopsis* (Penfield *et al*., 2010). One isoform with an intron retention (Fig. S4B) was significantly (*P *<* *0.01) downregulated in AGSL101 compared to that in A12DHd during seed development.

In *Arabidopsis* the second gene *At3g01060* currently is classed as a protein of unknown function. However, in a study demonstrating that PIF4 has a central role in the positive control of genes mediating hypocotyl cell extension, *At3g01060* was identified as one of several genes that were significantly repressed (De Lucas *et al*., 2008). The insertion mutants of *At3g01060* and *AtPTB1*, and also transformations expressing *B. oleracea* alleles for both genes had WT ABA contents; our results in both *Arabidopsis* and *B. oleracea* are therefore consistent with a role for *At3g01060* (*BoLCVIG1*) as an ABA effector altering sensitivity to ABA. Interestingly, transformation with the GDDH33 allele of *BoLCVIG1* increased sensitivity to ABA compared to transformation with A12DHd alleles. Nevertheless, ABA content influences speed of germination and we show that this is determined by *RABA1*. Furthermore we provide evidence that a homologue of Arabidopsis *CYP707A2*, a key ABA catabolic gene (Kushiro *et al*., 2004), present at this locus is the most likely candidate gene that determines ABA content.

### Proposed model for the regulation of seed vigour by *SOG1* and *RABA1*

**Figure 7:**
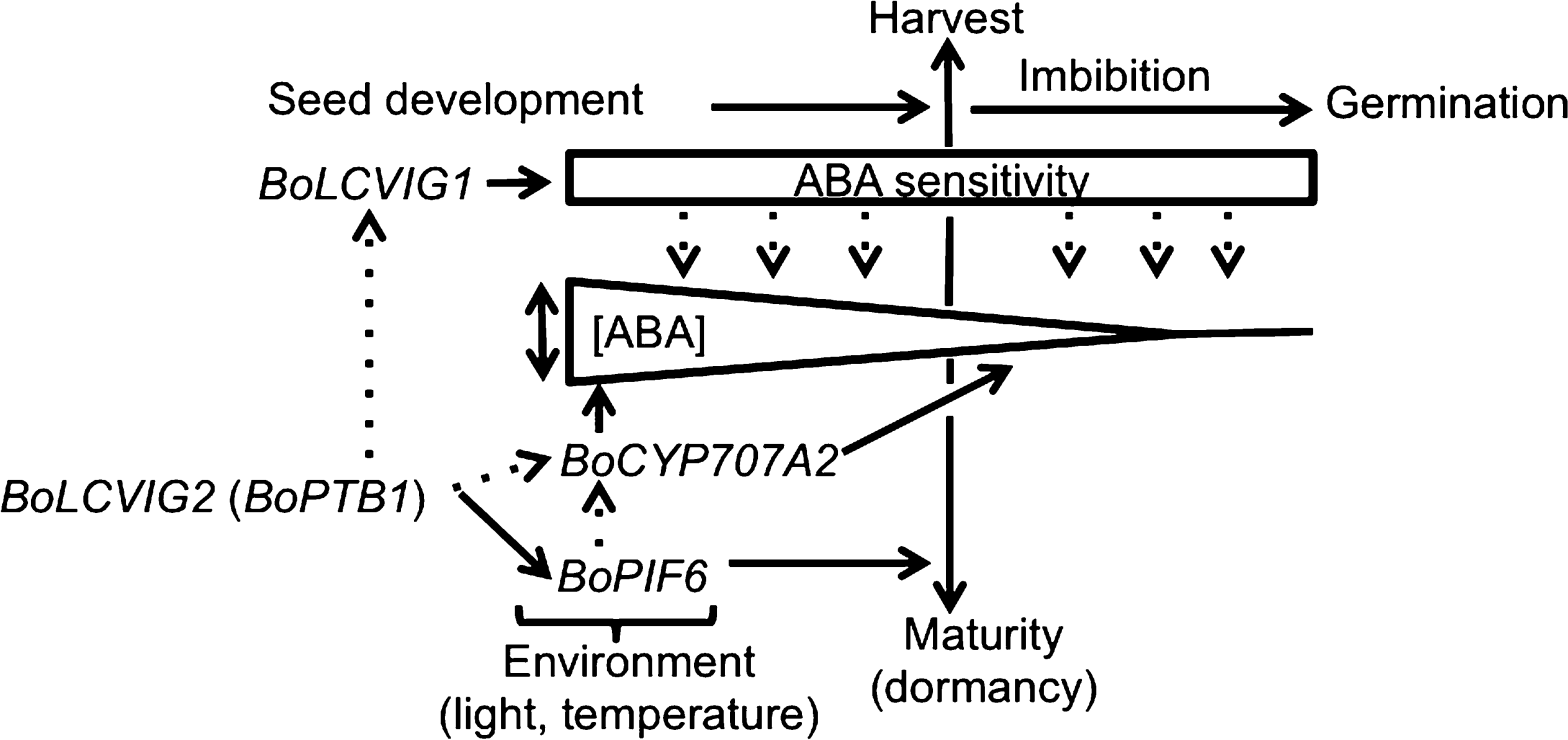
Schematic model for the regulation of *Brassica oleracea* germination by genes at *SOG1* (*BoLCVIG1* and *BoLCVIG2*) and *RABA1* (*BoCYP707A2*).

A model for the potential regulation of germination by genes in *SOG1* and *RABA1* is outlined in Fig. 7. In the model we propose that the progressive decline in endogenous ABA content towards the end of seed development is determined by the catabolic action of *BoCYP707A2* in *RABA1*, which is greater in AGSL101 (GDDH33 alleles) than A12DHd. We suggest that sensitivity to the ABA remaining during imbibition determines the speed of germination and that this sensitivity is determined by allelic variation in *BoLCVIG1* consistent with its role as a negative regulator of germination. Although further work is required to determine a causal link, *BoLCVIG2* (*BoPTB1*) has the potential to regulate alternative splicing (AS) in *BoLCVIG1*,* BoPIF6* and *BoCYP707A2* during seed development. We found no evidence of AS in *BoCYP707A2*, but demonstrate that *BoLCVIG1* exists in different splice forms and that their expression differs between seed development and imbibition and also between genotypes (Fig. 4).

Seed vigour and performance are complex traits that have remained difficult to define and influence at a genetic level. The isolation of genes in *SOG1* and a candidate in *RABA1* that are responsible for natural variation and the impact of allelic variation, provides a way forward to understand and manipulate the genetic pathways regulating this key agricultural trait. All three genes are highly conserved across species indicating the possible conservation of the mechanism proposed.

## Author contributions

W.E.F‐S., G.C.B. and K.M. conceived the experiments; K.M., G.C.B. and W.E.F‐S. performed the experiments; P.G.W. and J.R.L.; analysed data; W.E.F‐S., K.M. and G.C.B. wrote the manuscript.

## Supporting information

Please note: Wiley Blackwell are not responsible for the content or functionality of any supporting information supplied by the authors. Any queries (other than missing material) should be directed to the *New Phytologist* Central Office.

**Fig. S1** *B. oleracea* seed germination and hydrothermal time analysis.
**Fig. S2 **Schematic of the steps taken to identify candidate genes underlying the *SOG1* QTL in *B. oleracea*, which employed resources from both *B. oleracea* and *Arabidopsis*.
**Fig. S3 **The results of genotyping and phenotyping *B. oleracea* BILS to fine‐map SOG1.
**Fig. S4 **A summary of different isoforms identified in *BoLCVIG1* and *BoPIF6*.
**Fig. S5 **The *RABA1* locus.
**Table S1 **Summary of the 12 *Arabidopsis* orthologous gene models in the *B. oleracea* annotated BAC BoB064L23
**Table S2** *Arabidopsis* insertion mutants
**Methods S1 **Molecular biology experiments, protocols and data analyses.
