# Supplementary material for: Trait to gene analysis reveals that allelic variation in three genes determines seed vigour
Source: New Phytol. 2016 Jul 19;212(4):964–76. doi: 10.1111/nph.14102 (PMC5132119; doi:10.1111/nph.14102)
Supplement: Supplementary file 1 — Fig. S1 B. oleracea seed germination and hydrothermal time analysis. Fig. S2 Schematic of the steps taken to identify candidate genes underlying the SOG1 QTL in B. oleracea, which employed resources from both B. oleracea and Arabidopsis. Fig. S3 The results of genotyping and phenotyping B. oleracea BILS to fine‐map SOG1. Fig. S4 A summary of different isoforms identified in BoLCVIG1 and BoPIF6. Fig. S5 The RABA1 locus. Table S1 Summary of the 12 Arabidopsis orthologous gene models in the B. oleracea annotated BAC BoB064L23 Table S2 Arabidopsis insertion mutants Methods S1 Molecular biology experiments, protocols and data analyses. [file NPH-212-964-s001.pdf]

New Phytologist Supporting Information

Article title: **Trait to gene analysis reveals that allelic variation in three genes determines seed vigour**

Authors: Karl Morris, Guy C. Barker, Peter G. Walley, James R. Lynn and William E. Finch-Savage

Article acceptance date: 5 June 2016

The following Supporting Information is available for this article:

Fig. S1 *B. oleracea* seed germination and hydrothermal time analysis

Fig. S2 Schematic of the steps taken to identify candidate genes underlying the *SOG1* QTL in *B. oleracea*, which employed resources from both *B. oleracea* and *Arabidopsis*

Fig. S3 The results of genotyping and phenotyping *B. oleracea* BILS to fine-map SOG1

Fig. S4 A summary of different isoforms identified in *BoLCVIG1* and *BoPIF6*

Fig. S5 The *RABA1* locus

Table S1 Summary of the 12 *Arabidopsis* orthologous gene models in the *B. oleracea* annotated BAC BoB064L23

Table S2 *Arabidopsis* insertion mutants

Methods S1 Molecular biology experiments, protocols, and data analyses

Fig. S1 *B. oleracea* seed germination and hydrothermal time analysis. (a) Cumulative germination curves of seeds from four substitution lines AGSL101, AGSL111, AGSL118, AGSL119 and parent lines (A12DHd and GDDH33) at 15°C on water. Vertical lines are standard errors. (b) The impact of temperature and water stress on germination can be modeled using hydrothermal time (HTT) approaches and can therefore be predicted. Germination of AGSL101, GDDH33 and A12DHd seeds was recorded at three water potentials (0, -0.41, -0.79 MPa) and three temperatures (5, 15, 20°C) and the data were subjected to HTT analysis. In all three genotypes, germination rate decreased at lower temperatures and at lower water potentials. Linear regression lines were fitted to this response and in all three genotypes > 90% of the variance could be accounted for. The lines were compared by regression analysis. In the case of the response to water potential, lines could not be constrained to a single threshold and had a significantly (P<0.001) better fit when parallel lines were fitted and this was not improved by allowing the slopes to alter from each other. In contrast, with the temperature response the fit of lines was significantly (P<0.001) improved when the slopes of lines were allowed to be different and 96% of the variation could be explained when they were constrained to a single threshold. No more variance could be explained by allowing the threshold to differ. The data therefore conformed to the basic hydrothermal time model (i.e. a single temperature threshold, but a distribution of water potential thresholds) and so model parameters were fitted and optimized by minimizing the residual sum of squares of the differences between measured and modeled values. The greater effect of lower water potentials on A12DHd seed germination compared to AGSL101 illustrated in Fig. 1(c) is generalized in its higher $\Psi_b(50)$ (base water potential of the 50th percentile). $\Psi_b(50)$ is a measure of the sensitivity of germination to decreased water potential, the more negative $\Psi_b(50)$ for AGSL101 and GDDH33 than that of A12DHd indicates their greater tolerance to water stress and their greater speed of germination.

(a)

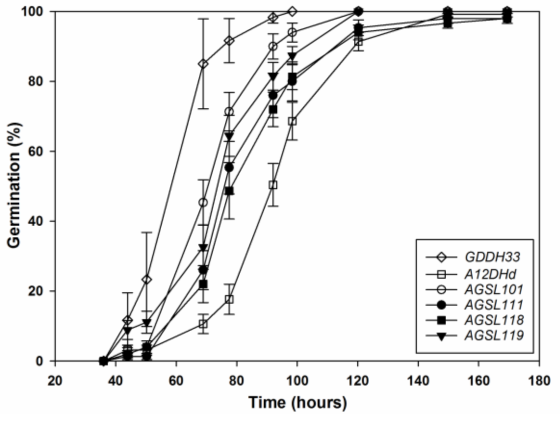

(b)

**Model parameters**	**GDDH33**	**AGSL101**	**A12DHd**
T_b (°C)	2.1	1.4	0.4
θ_{HT} (MPa °C day)	32	36	40
$\Psi_b(50)$ (MPa)	-1.5	-1.2	-0.6
$\sigma_{\Psi b}$ (MPa)	0.34	0.58	0.34

Fig. S2 Schematic of the steps taken to identify candidate genes underlying the *SOG1* QTL in *B. oleracea*, which employed resources from both *B. oleracea* and *Arabidopsis*. Crucial to this was the development of *SOG1* specific markers in *B. oleracea* to establish synteny with the upper arm of *Arabidopsis* chromosome 3. These markers needed to be polymorphic between A12DHd and GDDH33 alleles to define the GDDH33 introgression in the A12DHd background of the substitution line AGSL101 and the recombinations in this region present in Backcross Inbred Lines (BILs). This process involved the confirmation of the relevant synteny between *B. oleracea* and *Arabidopsis*, *B. oleracea* bacteria artificial chromosome (BAC) identification, BAC tiling path construction and candidate gene identification described below. This was followed by fine-mapping using *B. oleracea* BILs (Fig. S3) and screening of insertion mutants in *Arabidopsis* (Table S2)

Confirmation of the synteny between B. oleracea chromosome 1 and the top arm of chromosome 3 of Arabidopsis: More rapid germination of substitution line AGSL101 than the A12DHd recurrent parent line confirms that an introgressed region at the bottom telomeric end of chromosome 1 contains the QTL for *SOG1* identified by Bettey *et al.* (2000). Previously synteny has been shown between a number of regions in the *Brassica* C genome and *Arabidopsis* using the RFLP markers pR85 and pN13 (Cogan *et al.*, 2004). To develop further markers we designed primer pairs to 30 *Arabidopsis* gene models that were spaced across the region in *Arabidopsis*. These primers were tested to determine if they amplified a *B. oleracea* product and then if there was any polymorphisms between AGSL101 and the parental lines A12DHd and GDDH33. A banding pattern that is the same in AGSL101 and GDDH33, but different from that in A12DHd indicates its presence at the *SOG1* locus in *B. oleracea* and therefore its usefulness in refining the QTL. Primers for three gene models were identified as informative markers (*At3g01190*, *At3g02420*, *At3g07130*). In addition, public databases (Brassica.info-SSR exchange) were also screened for BoLGC1 markers that might be informative. A number of SSRs were identified and tested for any polymorphism between the *B. oleracea* lines and appropriate locations on *Arabidopsis* chromosome 3. Two SSR markers proved informative (Ni4B10 and OL10F10). These five markers confirmed the synteny between the top arm of *Arabidopsis* chromosome 3 and the *SOG1* region of *B. oleracea* chromosome 1.

Bacteria artificial chromosome (BAC) tiling path construction and BAC identification: Parallel to the above work to establish synteny we constructed a BAC (Bacteria Artificial Chromosome) tiling path spanning the introgressed region in AGSL101. The molecular markers were used to isolate BAC constructs from the BoB and JBo libraries (www.brassica.info/resources). GDDH33

genotype scores in the AGSL101 substitution line identified the chromosome 1 position of these BACs. A number of the BACs from this region were sequenced and annotated in relation to the *Arabidopsis* genome. During the process five polymorphic gene markers (Marker 1 = *At3g03110*; Marker 2 = *At3g02920*; Marker 3 = *At3g02555*; Marker 4 = *At3g02090*; Marker 5 = *At3g01150*) were selected to screen Backcross Inbred Lines (BILs) for fine mapping *SOG1*. The following primers were used: Marker 1 F: CACAGTGGCCTCTTGAACG, R: TCCAAACGAGATTCAAGACGA; Marker 2 F: GTACCTTGCTTCCCCTGACG, R:CTTGACTGAAATTGACTCGGTAGA; Marker 3 F: AACACGACGGGGCTTAGGAC, R: TTCGTTGGCTTATTCTGTTTTTG; Marker 4 F: GCTCAAGAGGCTGGATAACCC, R: CTCCCTCGACGTATACGCATTC; Marker 5 F: GTGGGAATGCGTGGAAGAGGAG, R: CAAAGGTGACCAAGAGGACATT.

References

Bettey M, Finch-Savage WE, King GJ, Lynn JR. 2000. Quantitative genetic analysis of seed vigour and pre-emergence seedling growth traits in *Brassica oleracea* L. *New Phytologist* **148**: 277–286.

Cogan NOI, Newbury HJ, Oldacres AM, Lynn JR, Kearsey MJ, King GJ, Puddephat IJ. 2004. Identification and characterization of QTL controlling *Agrobacterium*-mediated transient and stable transformation of *Brassica oleracea*. *Plant Biotechnology Journal* **2**: 59–69.

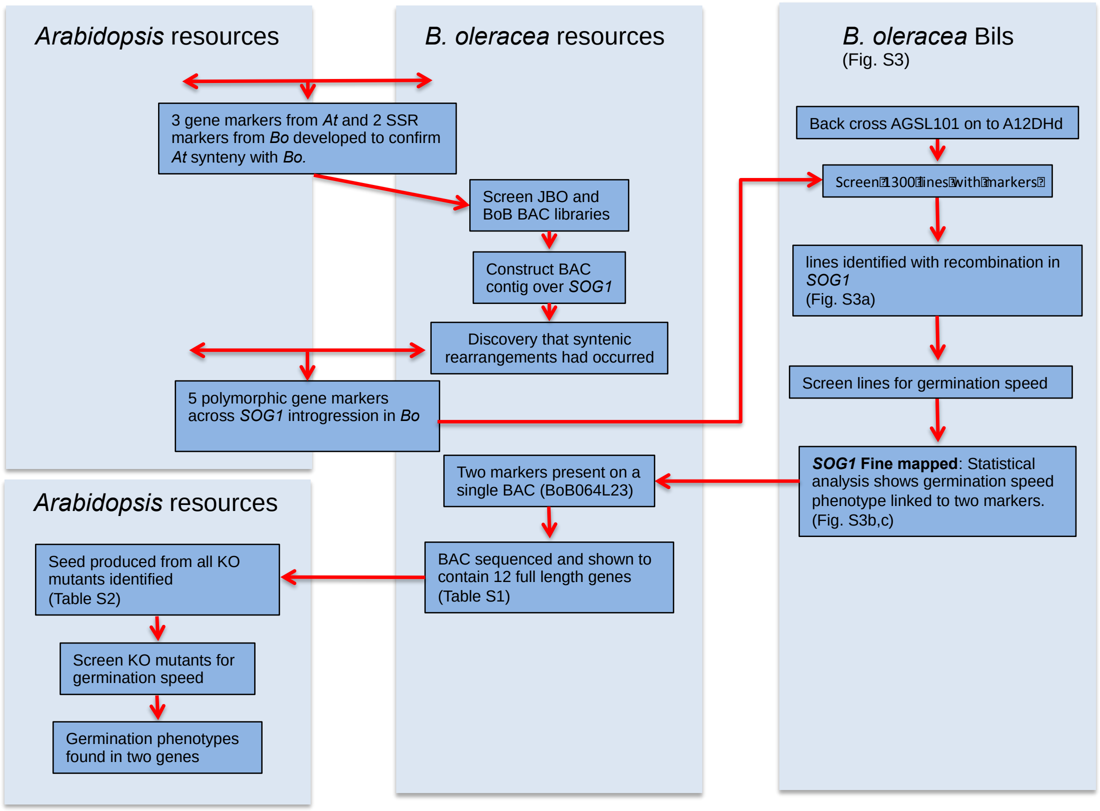
Arabidopsis resources
B. oleracea resources
B. oleracea Bils
(Fig. S3)
3 gene markers from At and 2 SSR markers from Bo developed to confirm At synteny with Bo.
Screen JBO and BoB BAC libraries
Construct BAC contig over SOG1
Discovery that syntenic rearrangements had occurred
5 polymorphic gene markers across SOG1 introgression in Bo
Back cross AGSL101 on to A12DHd
Screen 1300 lines with markers
lines identified with recombination in SOG1
(Fig. S3a)
Screen lines for germination speed
SOG1 Fine mapped: Statistical analysis shows germination speed phenotype linked to two markers.
(Fig. S3b,c)
Two markers present on a single BAC (BoB064L23)
BAC sequenced and shown to contain 12 full length genes
(Table S1)
Arabidopsis resources
Seed produced from all KO mutants identified
(Table S2)
Screen KO mutants for germination speed
Germination phenotypes found in two genes

Fig. S3 The results of genotyping and phenotyping *B. oleracea* BILS to fine-map *SOG1*. (a) schematic of the *SOG1* region in selected BILs (F_6) identified as having recombination in the genotype screen. This recombination is indicated by the genotype scores (A12DHd or GDDH33) from the five polymorphic markers (Fig. S2) used in the screen. (b) The lines were also subjected to a phenotype screen at the low temperature of 8°C to enable more recordings to be made and thus an accurate cumulative germination curve. Speed of germination was calculated as mean log time to germination as used by Bettey *et al.* (2000) in the original analysis that identified *SOG* QTL. These data were used to refine *SOG1* through statistical analysis with the genotype scores. The analysis considered each marker, or each pair of consecutive markers, separately and determined if there was any associated difference. The analysis was performed using REML, taking the marker score or scores as fixed, and variability between lines with the same marker score(s), and between replicates as random. It was anticipated that for some groups of lines with the same marker score, the QTL would not be segregating, and for others that it would, so the variance component estimating variance between lines with the same marker score was allowed to vary between marker scores. In this single marker analysis the speed of germination is significantly different (P<0.05) with GDDH33 (G) alleles at markers 4 and 5 with increased significance at marker 5 to when A12DHd (A) alleles were present. **(c)** This result was substantiated in the between marker analysis with the only significant difference being observed between markers 4 and 5. The analysis was repeated using germination data from the F_5 generation and this confirmed the result. The *SOG1* QTL was therefore fine mapped to this region identified by markers 4 and 5.

It should be noted that in the lines analyzed, if a QTL is significant for GDDH33 at marker five the results will also show significance for the reverse effect i.e. A12DHd at marker 1 (as in S3b). This opposite significance effect is an artifact of recombination occurring at the end of the chromosome (chromosome all A12DHd to the start of the introgression) so that almost all lines with recombination will have GDDH33 at marker 5 and A12DHd at marker 1 (see schematic S3a). Thus lines with faster germination due to GDDH33 alleles at marker 5 will also almost all have A12DHd at marker 1.

Further genotype screening and analysis show that genes associated with markers 4 and 5 separately influence germination speed: Subsequently other gene markers were developed for several of the genes identified on the BAC BoB064L23 (Table S1). These included *At3g02080* and *At3g01060 (BoLCVIG1*) grouped with marker 4 and *At3g01070* and *At3g01090* grouped with marker 5 (*BoLCVIG2*). The BILs were genotyped again using these new markers and we found

that a recombination event identified with markers 4 and 5 had occurred between the two genes *BoLCVIG1* and *BoLCVIG2* so that they were grouped with marker 4 (*Bo3g02090*) and 5 (*Bo3g01150*) respectively. The analysis for speed of germination of the BILs described above showed it was significantly different at markers 4 (includes *BoLCVIG1*) and 5 (includes *BoLCVIG2*). Thus genes associated with both markers independently influence *SOG1*. We then extended the statistical analysis to look for linkage at pairs of markers and found that markers 4 and 5 were significantly linked ($P<0.012$) suggesting that genes associated with these markers influence speed of germination in the same way. The primers for the additional markers were: *Bo3g02080*, F: ATGGCAACTGGTAAAAC, R:TCAGAAGAATGGATATAGAAGATG; *Bo3g01060*, F: TGGCTCGTCGCATACAACAAAG, R: TAGCGCGAAAGATACCGAAAAACT; *Bo3g01070*, F: ATGGCTAGACTTGCGTTGATGG, R:TCTTGATAGGTGCTGCGATTGGTG; *Bo3g01090*, F: GCTTGGCAGAACTCTCGGTATCG, R: AGTGCCCCCAAATGAAGAAGAGG.

(a) Result of the genotype screen

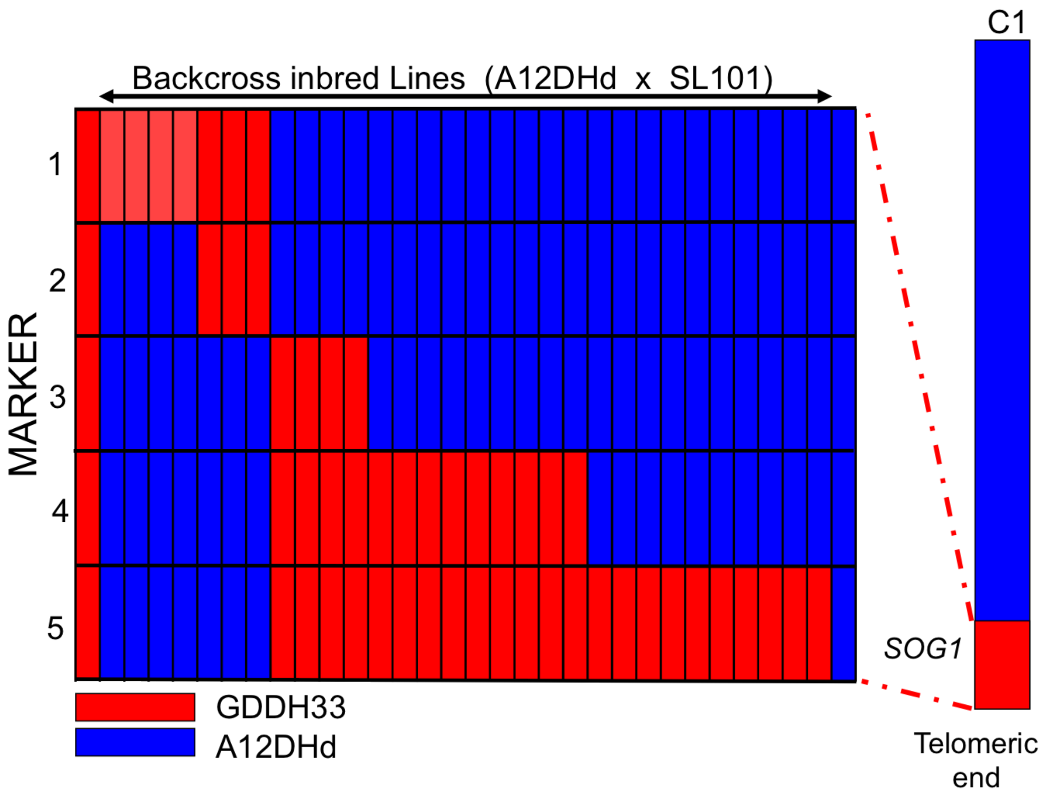

(b) Result of the phenotype screen for speed of germination (single marker). A = A12DHd, G = GD33DH; Data are mean log time (hours) to germination.

MARKER	*P* value (*F* test)*	A	G
**1. At3g03110**	0.003	1.68	1.76
**2. At3g02920**	0.066	1.693	1.749
**3. At3g02555**	0.327	1.708	1.67
**4. At3g02090**	0.024	1.729	1.669
**5. At3g01150**	0.002	1.778	1.667

(c) Result of the phenotype screen for speed of germination (between markers). A = A12DHd, G = GD33DH; Data are mean log time (hours) to germination.

MARKERS	*P* value (*F* test)*	AA	AG	GA	GG
**1 and 2**	0.011	1.68		1.766	1.749
**2 and3**	0.262	1.702	1.636	1.755	1.739
**3 and 4**	0.121	1.279	1.669		1.67
**4 and 5**	0.004	1.778	1.688		1.669

Table S1 Summary of the 12 *Arabidopsis* orthologous gene models in the *B. oleracea* annotated BAC BoB064L23

**Predicted gene no.**	***Arabidopsis* ortholog**	**Putative function**
1	*At3g02200*	Proteasome family protein
2	*At3g02090*	Mitochondrial processing peptidase
3	*At3g02080*	40S ribosomal protein S19
4	*At3g01060* (*BoLCVIG1*)	Unknown
5	*At3g01070*	Plastocyanin-like domain-containing protein
6	*At3g01085*	Protein kinase family protein
7	*At3g01090*	AKIN10 (Arabidopsis SNF1 kinase homolog 10)
8	*At3g01150* (*BoLCVIG2b*)	PTB1 (Polypyrimidine Tract-Binding protein 1)
9	*At3g01140/42*	MYB106 (myb domain protein 106) or naturally occurring antisense RNA
10	*At3g01150* (*BoLCVIG2a*)	PTB1 (Polypyrimidine Tract-Binding protein 1)
11	*At3g01175*	Protein unknown function DUF1666
12	*At3g01180*	Starch Synthase

Table S2 *Arabidopsis* insertion mutants. (a) Orthologous *Arabidopsis* genes and insertion lines available for genes located on *B. oleracea* BAC BoB064L23. [a] *Arabidopsis* AGI in the order they are located on chromosome 1, from the centromere towards the bottom telomeric end. [b] Summary of the T-DNA insertion KO lines obtained for germination testing. [c] = FLAG mutant could not be genotyped. In experiments comparing all insertion lines and wild type controls only four lines (*) had significant germination phenotypes (Fig. 2). (b) Schematic diagrams of the insertion sites in these four lines. Insertions were verified by PCR using the primers for the different lines as recommended by the T-DNA express site and the T-DNA primer Lb1.3 (ATTTTGCCGATTTCGGAAC). Primer sequences SALK_100860 LP TTTTCCATCATAGCTTCCACG, SALK_100860 RP ACCTGGACACACTCTTTGGTG; SALK_009486 LP GCTCCAAAGAATTGTTGAATGAC, SALK_009486 RP AAAATAGCTCGCAGCTTTTCC; SALK_013673 LP AGTTTCTGCATATGCTGCCAG, SALK_013673 RP GTAAGGAGGTTGACCTGAGGG; SALK_107494 LP CAATGGAAACAAATTGAATTTGTG, SALK_107494 RP TCAATAGAAATCGCCAAAACG.

(a)

Arabidopsis AGI[a]	Insertion ID[b]
At3g02090	No insertion mutants [c]
At3g02080	No insertion mutants
At3g01050	No insertion mutants
At3g01060	SALK_009486*
	SALK_034607
	SALK_100860*
At3g01070	No insertion mutants
At3g01085	SALK_114870
	SALK_120335
	SALK_073654
	SALK_100754
	SALK_120095
At3g01090	SALK_127939
	GABI-KAT 579E09
At3g01200	SALK_035652
	SALK_035653
At3g01140/42	SALK_025449
	SALK_110059

At3g01150	SALK_107494*
	SALK_013673*
At3g01175	No insertion mutants
At3g01180	SALK_065639
	SALK_102650

(b)

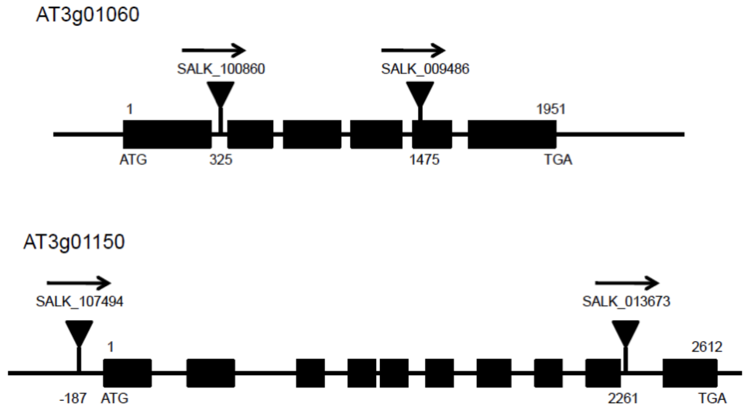

Fig. S4 A summary of different isoforms identified in *BoLCVIG1* and *BoPIF6*. The *y* axis shows the normalised uniquely mapable read coverage (read depth). The gene structure (*x* axis) is shown as in Fig. 4. (A) *BoLCVIG1* reads derived from RNAseq data of replicate samples (1 and 2) taken from both A12DHd and AGSL101 seeds during development (D) and imbibition (IB) mapped on to the A12DHd sequence of the *BoLCVIG1* gene. The starred region shows reads in A12DHd that clearly span an intron supporting the evidence for isoform D specific intron retention within the spliceforms present. (B) An example of the alternative splice forms in *BoPIF6* present during seed development. The starred region indicates where intron retention has occurred.

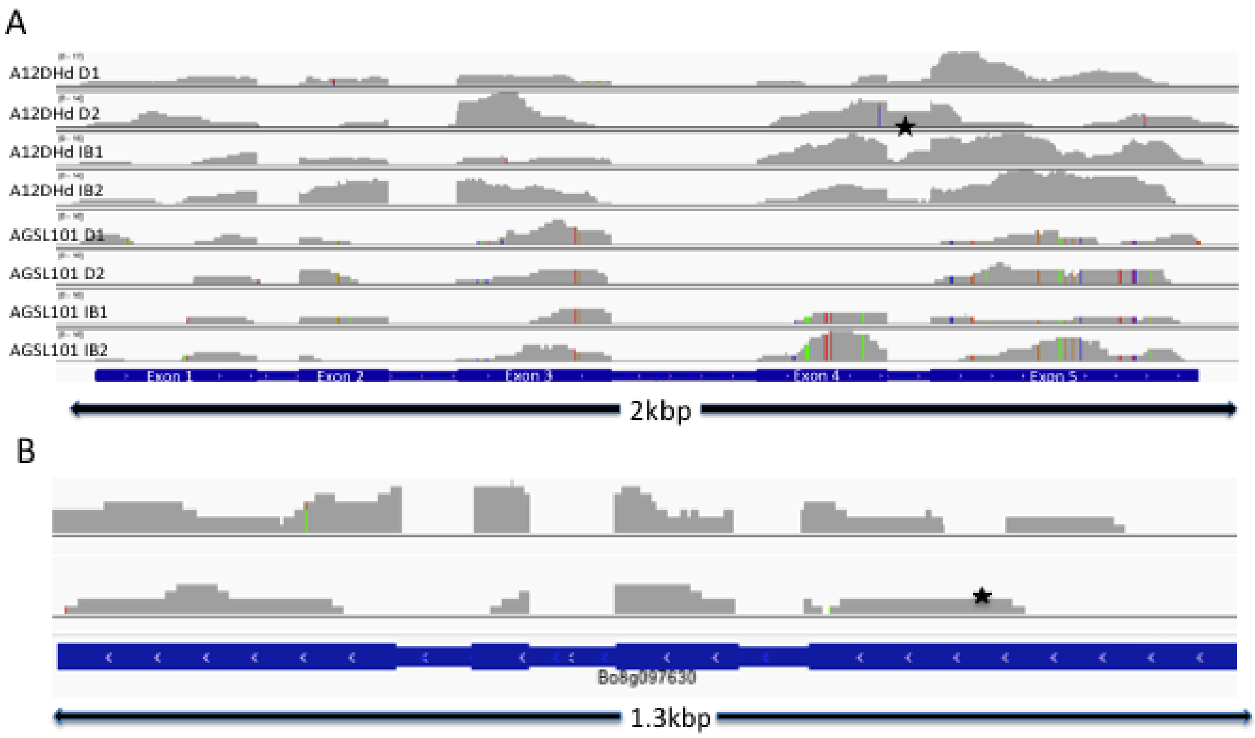

Fig. S5 The *RABA1* locus. (a) The relationship between mean T50 and ABA content in *B. oleracea* BILs (A12DHd X AGSL101). Dotted line is fitted to all data ($r = 0.411$, df 33, $P<0.05$). (b) A section of *B. oleracea* chromosome 4 genotyped with 10 markers (A–J) for the collection of BILs, and the A12DHd and AGSL101 parents. ABA content is shown on the right hand side indicating two clear groupings (lower and higher ABA content (> or < 300 ng ABA g^{-1} DW). Lines with lower ABA content had been assigned GDDH33 (yellow) by the markers and those with the higher ABA content had been assigned as A12DHd (Blue) at 4 markers (G–J) that define the *RABA1* locus. White indicates where markers could not be clearly assigned. Marker J indicates the end of the GDDH33 introgression in AGSL101. The BILs were genotyped using the following SNP markers (A–J):

Marker A:

CTTTCAACCCTGTTATAATCTCAGCAAGAACCACTCCGAAGCTGTAGAC[G/A]TCGCTC
TTATCRGAGAGATGAAAGCATTGATGGTACTGCGGGTCGAGGTA

Marker B:

GATTATTTAAACAAATTTGTCTGCGATTTCCATGAAGTGATCACAGAGT[T/G]GCTGAG
GCTTGCAGAACATCGGCATGTGATCTGTCTCGTCTATCTCCAT

Marker C:

GGGTTCTGAGAAACGACTGTGGATATCTTCTGAGGAAGAACCGCACGAA[G/T]CCCAG
ATATGCAGTGAGTCGAGGCGAGGCTCGAGGAGCTGAATCGAGCGA

Marker D:

ACCAAGTTCATGAAGCCTACTTTGTGATCCAAGAAACTGCCAAAGCGGC[G/C]AAAAC
AACATCTGACAGAGCTTATCTAACCTACTTATGTGCGCAAGAAAT

Marker E:

AACACAGCTTAGAAACAATTACTCTCCTGCTTGCGTTAAAGGTAGTTTT[T/C]AGAGCA
CCATATTAATGATATCTCTTTTCCGGTATTCTTGATAGAAATGG

Marker F:

TCAGCTAAKATACCATACAGTTCTGCAATCGGAGTCCCTGATTTCAGAG[A/C]GTTGA
ATAGGATAAGAGAATCYGTGTTGCAGTGTAGTCGCTTAGTATCCC

Marker G:

ACTAGCCGTAGAGATAAGGAACTATTGGCGCAACTATTGTTCCGTGGAG[G/T]TAGTA
ACGGCGGAAGAGATAGAGAGAGCAATCACGTGTTTGATGAAGCAG

Marker H:

AGATGTCAAGGAAACTGCGGTTTTTCAAAGATCAAATTGATAAAGCTGG[C/T]CTAAG
ATGTTTACCACGCCATGAATTAGAACCGGACATTGAGCTTGGCGA

Marker I:

AGTGAGGCCTAGGAGGGTTGCACCATCCGCCATTGTCACTAGGCTGAGC[G/A]AAGTT
TGGAGGRCAGAAGTTGGTSGCAGTGACGAAGATGGAAGGGCTACC

Marker J:

CTTCGCCGAGATTCAAACCCTTGAGGCCAACGAGATCAGAGAATTTGAC[T/G]TTATC
TTGAAAGGGAACTTTAATCATTCAGGTTTTAGCCCTACCAAGTTA

These markers were identified following genomics NGS sequencing to a depth of 50 X coverage of both A12DHd and GDDH33 followed by alignment to the TO1000DH reference genome (Parkin *et al.*, 2014) and SNPs identified using both SAMtools and SOAPsnp. Markers were designed at intervals of approx. 10,000 bp. (c) A summary of RNAseq data showing de-regulated genes at the *RABA1* locus of chromosome 4 from *B. oleracea*. Fold change is the difference in expression between AGSL101 relative to A12DHd expression. No other genes at this locus showed a significant difference between genotypes.

(a)

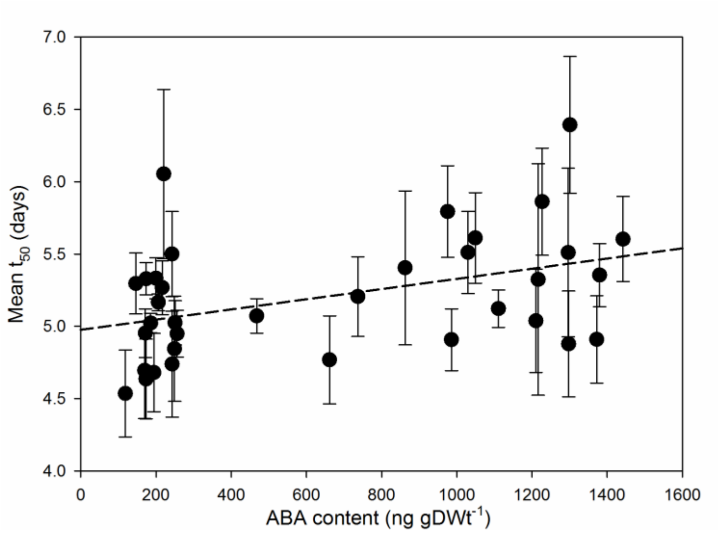

(b)

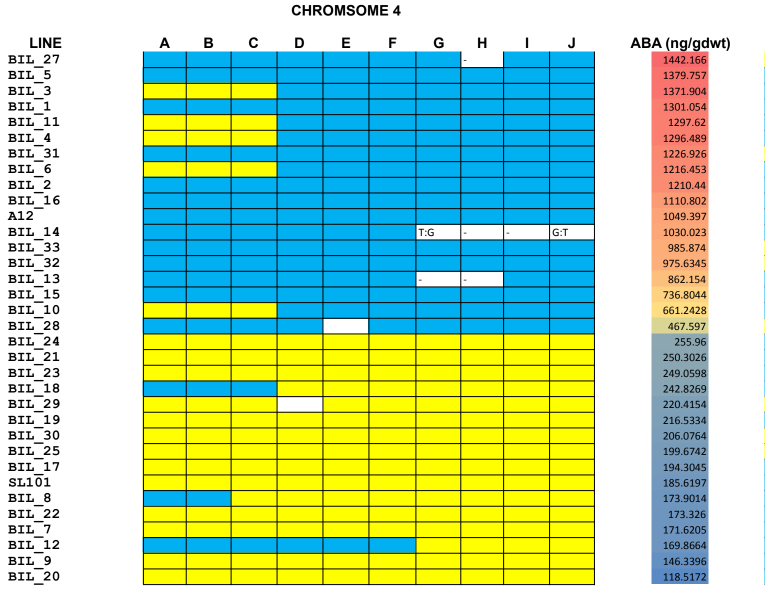

(c)

**Bo Gene ID (Gene name)**	**RNAseq library**	**Fold change**	**Log$_2$fold change**	***P* value**
Bo4g167920 (Expansin-A6)	Imbibition	5.25	2.39	6.39E-07
Bo4g168080 (*CYP707A2)*	Imbibition	3.81	1.93	1.27E-09
Bo4g165050 (Exostosin family protein-like protein)	Development	0.19	-2.42	7.17E-11
Bo4g168080 (*CYP707A2)*	Development	46.5	5.54	3.57E-15
Bo4g169180 (Rossman-fold superfamily protein)	Development	4.07	2.03	1.42E-06

Methods S1

Marker analysis to confirm collinearity between *B. oleracea* and *Arabidopsis*

Primer pairs were designed to 30 *Arabidopsis* gene models that were spread at intervals across the *SOG1* region using Primer 3 software (http://gene.pbi.nrc.ca/cgi-bin/primer/primer3_www.cgi) and gene data from TAIR (http://www.arabidopsis.org/) to give amplicons from 200 to 700 bp. The PCR mix used was standard, but a touch-down program was used. This consisted of cycling parameters as follows: 94ºC for 5 mins; then annealing at 65ºC to 55ºC for 10 cycles dropping a degree each cycle with 30 s extension at 72ºC and 30 s denaturation at 94ºC over the 10 cycles; followed by 30 cycles of 94ºC 30 s, 55ºC 30 s, 72ºC 45 s; and a final extension at 72ºC for 15 min. A number of publically available SSRs used in the generation of the *B. oleracea* integrated map (Sebastian *et al.*, 2000) were also used. Two proved informative (Ni4B10 and OL10F10).

Marker analysis used to genotype BILs

Hybridisation of the markers designed above was used to screen an A12DHd BAC library (Howell *et al.*, 2002) positive clones were re-probed with adjacent markers. BACs with numerous hits were end sequenced and promising BACs fully sequenced. Further probes were identified from the sequences obtained and these were used to re-probe the BAC library. The process was then repeated several times and a BAC tiling path generated. Locus specific primers were then designed. These were tested using the PCR conditions shown above. PCR amplicons for sequencing were cloned and at least three clones sequenced using standard protocols using the Big-Dye Terminator system (Applied Biosystems, Warrington, UK) with products run on an ABI Prism 3130xl Genetics Analyzer (Applied Biosystems). Primers that gave polymorphic results were selected as markers (Fig. S2). These were used to fine map the QTL within the backcrossed lines generated.

Transgenic constructs

Full sequence of the *B. oleracea* homologues (*BoLCVIG1* and *BoLCVIG2*) of both genotypes have been submitted to GenBank (AC. KM046981, KM046983, KM046984, KM046985). *Arabidopsis* accession Shakdara (Sha) was transformed. For *BoLCVIG1* alleles *a* complementary DNA fragment including the full length open reading frame (OFR) and 1.2 Kb of promoter for both A12DHd and GDDH33 alleles were PCR amplified using KOD Taq polymerase (Novagen) with primers (*BoLCVIG1* F (CACCAAGAGTGTAAGCGACCCATTTCAA) and *BoLCVIG1* R (TTGTTCCGTCATCTTCGTTTTCTA) and cloned into the pENTR/D-TOPO vector (invitrogen)

and inserted into the pMDC123 destination vector. For *BoLCVIG2* alleles the full length ORF and approx. 2.2 Kb of promoter was used. *BOLCVIG2* alleles were amplified with *BOLCVIG2* F (CACCTAAAAGTAAACAAAAAGAAGGAAC) and *BOLCVIG2* R (AAAGTCAAATAGAAACAAATACACAATG) primers. The pENTR/D-TOPO entry vector was inserted into the pMDC123 destination vector.

RNA extraction

Seeds were selected at random from bulked samples for RNA extraction. Total RNA was extracted in lots of 12–15 seeds and then RNA was pooled from at least 50 seeds in each replicate sample. RNA samples were then further pooled depending on their use (see below). Extraction was by an RNAqueous kit (Ambion) in conjunction with Plant RNA isolation aid (Ambion). RNA quality was determined using a spectrophotometer (Nanodrop, USA) and electrophoretically with an Agilent Bioanalyser. RNA was treated with RNase-free DNase I (Roche Diagnostics) to remove contaminating genomic DNA.

Illumina sequencing and analysis

For Illumina sequencing RNA was combined across harvests to give two replicates of a single seed development and imbibition sample for both A12DHd and AGSL101. Oligo(dT) selection was performed twice using Dynal magnetic beads (Invitrogen). Both GAIIx and MiSeq sequencing was carried out on two biological and three technical replicates. Illumina library preparations were performed using mRNA-TruSeq sample prep kit version five (Illumina Inc., San Diego, CA, USA) according to the manufacturer’s protocol (15018818 revA). The cultivar/tissue specific libraries were randomly assigned to six-nucleotide multiplex barcoded adapters and lanes. Data was collected using short reads on an Illumina GAIIx instrument. 36 base paired-end sequence reads were base-called and scored for read quality using the Illumina CASAVA pipeline. MISeq sequencing was carried out using read lengths of 150 bp. Subsequently TopHat, Bowtie and Cufflinks were used to map the reads to both to the BAC tilling path assembled for this work and also to the TO1000DH (Parkin *et al.*, 2014) genomic sequence to give the number of mapped reads per kilobase of exon per million mapped reads (RPKM) a measure of transcript abundance. Splice form identification was carried out using Miso, MapSplice and DiffSplice programs allowing identification of both misplicing and intron retention.

PCR of alternative splice forms

Expression of alternative splice forms of *BoLCVG1* and *BoLCVG2* was determined by Illumina

sequencing as described above and was verified via PCR. The development and imbibition sample series described above were both pooled into early and late RNA samples. Synthesis of cDNA was performed on 2 μg total RNA using SuperScript^{TM} II Reverse Transcriptase (Invitrogen) and oligo dT primer (Invitrogen) following manufacturers' instructions. PCR was performed in 96-well plates with a Gold block PCR machine (Apllied Biosysyems). The gene specific-primers pairs used were: Primer 1 F (ATG GCG GCAGCGGCCATGGCCGTTC); R (ACACCCGCCAAAAAGGATGGG); and Primer 2 F (GGCTGATGAGATCGAGCAGAAAGTC); R (CTCCAGTCACTCGCTATCTCATCG). Reactions were performed in 5 μl of Master Mix (MgCl2, TAQ, dNTPS), 2 μl PCR-grade water, 1 μl of 25 mM forward and reverse primers and 1 μl cDNA (diluted 1:10) in a final volume of 10 μl. PCR were carried out under the following conditions: one cycle at 95°C for 3 min followed by 5 cycles Touchdown PCR -1C per cycle starting at 60°C followed by 30 cycles at 95°C for 30 s, 57°C for 30s, and 72°C for 30 s.

Quantitative RT-PCR

Expression of *BoCYP707A2* during seed development and seed imbibition to the completion of germination was determined by quantitative RT-PCR on the RNA from three replicate samples as described above. The qRT-PCR were performed in 384-well plates with a LightCycler® 480 Real-Time PCR instrument (Roche Diagnostics) using the LightCycler® 480 SYBR Green I Master kit (Roche Diagnostics). The gene specific-primers pairs used were F CAGGAGAGTGATGGATTCGTC and R TAAGAGCGGCAAGTGTATT. The housekeeping gene At4g34270 (Tip 41-like) was selected as a reference gene for normalization purposes and primers were prepared to a corresponding *Brassica oleracea* EST (qRT-Bo TIP 41-like). Forward was CACCGGAAGCCTCTGACTGAT and reverse TGATGGTGTGCTTATGAGGTTGAG. Reactions were performed in triplicate and contained 5 μl of SYBR Green I Master, 2 μl PCR-grade water, 1 ml of 10 mM forward and reverse primers and 1 μl cDNA (diluted 1:10) in a final volume of 10 ml. PCR were carried out under the following conditions: one cycle at 95 C for 10 min followed by 50 cycles at 95°C for 30s, 65°C for 30 s, and 72°C for 30 s. Data was analysed using LightCycler® 480 software (version 1.5; Roche Diagnostics). All qRT-PCR products were cloned into pGEM-T vector (Promega) and sequenced verified.

Data analysis

Sequence reads were aligned to the published *B. oleracea* genome assembly (Parkin *et al.*, 2014) using Tophat and Bowtie algorithms. Expression levels were calculated using TopHat, Cufflinks

and Cuffdiff to quantify expression differences. Normalisation was carried out using reads per kilobase of exon model per million mapped reads (RPKM counts). RPKM values are useful for analyzing differences in the abundance of alternative splice variants between samples, as correction for the length of each splice variant is essential for this type of analysis. MiSO, DEXSeq, and CuffDiff (implemented in the Cufflinks package) were all separately used to test for differences in alternative splicing between two samples. Counts of four or more were used to define gene expression. Visualization was performed by the Integrative Genome Viewer (IGV; Robinson *et al.*, 2011).

References

Howell EC, Barker GC, Jones GH, Kearsey MJ, King GJ, Kop EP, Ryder CD, Teakle GR, Vicente JG, Armstrong SJ. 2002. Integration of the Cytogenetic and Genetic Linkage Maps of *Brassica oleracea*. *Genetics* **161**: 1225–1234.

Parkin IAP, Koh C, Tang H, Robinson SJ, Kagale S, Clarke WE, Town CD, Nixon J, Krishnakumar V, Bidwell SL *et al.* 2014. Transcriptome and methylome profiling reveals relics of genome dominance in the mesopolyploid *Brassica oleracea*. *Genome Biology* **15**: R77.

Robinson JT, Thorvaldsdottir H, Winckler G, Guttman M, Lander ES, Getz G, Mesirov JP. 2011. Integrative Genome Viewer *Nature Biotechnology* **29**: 24–26.

Sebastian RL, Howell EC, King GJ, Marshal DF, Kearsey MJ. 2000. An integrated AFLP and RFLP *Brassica oleracea* linkage map for two morphologically distinct doubled-haploid mapping populations. *Theoretical and Applied Genetics* **100**: 75–81.
